# Supplementary material for: Construction of a competency evaluation index system for front-line nurses during the outbreak of major infectious diseases: A Delphi study
Source: PLoS One. 2022 Jul 1;17(7):e0270902. doi: 10.1371/journal.pone.0270902 (PMC9249240; doi:10.1371/journal.pone.0270902)
Supplement: S1 File — (DOCX) [file pone.0270902.s001.docx]

**Outline of an interview with nursing managers:**

1. Please briefly describe your main job duties during the anti-epidemic period.
2. Please talk about your experience and understanding of the characteristics of nursing work in isolation wards from your own perspective.
3. What knowledge, skills, abilities, and personal characteristics do you think nurses should have in order to be able to work in isolation wards during a sudden major infectious disease outbreak?
4. In addition to the above, what other competency characteristics do you think front-line nursing staff should have?

**Outline of an interview with nurses:**

(I) Please briefly describe your main job duties during the fight against the epidemic.

(II) Please describe three successful and three regrettable events that occurred during your front-line work against the epidemic.

1. What was this event? Why did it happen? What was the situation?

2. Who were the people involved in this incident? What did they do? What were your feelings?

3. What was the main task you faced at that time? To achieve what goal?

4. What were your thoughts and feelings at that time? What did you do afterwards? What was the result?

5. What do you think were the reasons for your success (or lack of success) in making this work?

(III) What knowledge, skills, abilities, and personal attributes do you think would allow you to remain successful in a front-line nursing position in the event of a sudden major infectious disease outbreak?

(IV) What are the challenges you face in fighting an epidemic? What knowledge, skills, abilities, and personal attributes do you need to develop to face these challenges?
