# Supplementary material for: Construction of a competency evaluation index system for front-line nurses during the outbreak of major infectious diseases: A Delphi study
Source: PLoS One. 2022 Jul 1;17(7):e0270902. doi: 10.1371/journal.pone.0270902 (PMC9249240; doi:10.1371/journal.pone.0270902)
Supplement: S2 File — (DOCX) [file pone.0270902.s002.docx]

**Construction of a competency evaluation index system for front-line nurses during the outbreak of major infectious diseases（the first round）**

Dear Experts:

I appreciate your willingness to participate in this study as a correspondence expert!

I am Xue Bai, a master's student of Professor Xiuni Gan at the Second Affiliated Hospital of Chongqing Medical University, and we are working on "I am Xue Bai, a master's student of Professor Xiuni Gan at the Second Affiliated Hospital of Chongqing Medical University, and we are working on "Development and the Test of Reliability and Validity of a Competency Quality Evaluation Model for Front-line Nursing Staff during the Outbreak of Major Infectious Diseases".

Based on the preliminary literature research and qualitative interviews, after the meeting of our expert group, a preliminary framework for evaluating the quality of front-line nursing staff competency during the outbreak of major infectious diseases was formed, including 4 primary indicators, 12 secondary indicators, and 67 tertiary indicators, which still need further guidance from experts in terms of the coverage of indicators and the importance of content.

The expert questionnaire is divided into two sections: the first is a fundamental information questionnaire, and the second is a questionnaire for evaluating the competency of front-line nursing personnel in large infectious disease epidemics, as shown in Tables 1-4. The information you give us will be kept private and used only for this study.

We truly hope to receive your advice and assistance, and your opinions will serve as an important basis for our research, given your outstanding academic achievement in this subject, your devotion to COVID-2019 or other infectious disease prevention work, and your extensive clinical expertise.

Two rounds of expert consultation are proposed for this study, with the first round of consultation to determine the content of each indicator and the second round of consultation to determine the weighting. Due to the timeliness of research and the progress of the study, we kindly ask that you respond to your comments and suggestions within 1 week. If you have any questions about the questionnaire, please feel free to contact us.

Thank you from the bottom of my heart for all of your help and advice. I wish you good work and good health.

The Second Affiliated Hospital of Chongqing Medical University

Tutor：Xiuni Gan

Postgraduate：Xue Bai

Contacts：Xue Bai Phone / Wechat：15761602836 E-mail：584454151@qq.com

Part 1: The expert basic information questionnaire

1. This questionnaire is designed to understand your situation. The information is only used for statistical analysis, absolute confidentiality, and never for other purposes. Please fill in the form according to your actual situation, and mark the "√" behind the indicator or mark the appropriate option in red. If you need further explanation, please mark the corresponding column.

| Name |  | Gender | □Male  □Female | | Age | ____year old |
| --- | --- | --- | --- | --- | --- | --- |
| Highest degree | □Doctor  □Master  □Undergraduate  □Specialty  □Other：____ | Research Directions | □Critical care medicine  □Emergency medicine  □Epidemiology  □Public Health  □Critical Care  □Emergency care  □Nursing Management  □Oteher：_____ | | Work Year | ____year |
| Position | □Director of the hospital □Deputy director of the hospital  □Head of department □Deputy head of department  □Head of the nursing department □Deputy head of the nursing department  □Head nurse of the department □Head nurse  □Lead teacher □Other：_____ | | | | Title | □Positive senior  □Deputy senior  □Intermediate  □Other：_____ |
| Whether the tutor | □Doctoral Tutor □Postgraduate Tutor □No | | | | | |
| Work Unit |  | | | | | |
| Telephone |  | | | E-mail | |  |

Part 2：Front-line nursing staff competence assessment indicators in the outbreak of major infectious disease questionnaire

Instructions for completing the form：

**一、**This section contains a total of 4 correspondence forms, of which：

Table 1: Primary indicators correspondence table；

Table 2: Secondary indicators correspondence table；

Table 3: Tertiary indicators correspondence table；

Table4: Please rate your judgment basis, degree of influence, and familiarity with the above items.

二、Please give your rating for the importance of each indicator based on your own experience and knowledge.

1.Importance score：Very important =5 points；More important = 4 points；General =3 points；Not very important = 2 points；Not important = 1 point, Please make a judgement on the relative importance of the indicator and tick the appropriate box.

2.If you believe that the description of the indicator is inaccurate or should be deleted, please fill in the "Comments for amendment or deletion" field or indicate "Delete". If you think there are additional indicators that we have not considered, please add them in the "Suggested additions" blank box, and please judge the importance of the changes and additions in the same way.

Table 1 Primary indicators correspondence table

Note：Importance score：Very important =5 points；More important = 4 points；General =3 points；Not very important = 2 points；Not important = 1 point

| Primary indicators | Importance score | | | | | Expert opinions |
| --- | --- | --- | --- | --- | --- | --- |
|  | 5 | 4 | 3 | 2 | 1 | Modify or delete comments |
| 1. Infectious disease knowledge system |  |  |  |  |  |  |
| 1. Infectious disease nursing skills |  |  |  |  |  |  |
| 1. Infectious disease response capacities |  |  |  |  |  |  |
| 1. Personal Traits |  |  |  |  |  |  |
| If you have suggested additional items, please fill in the blank lines below (note: please judge their importance) |  |  |  |  |  |  |
|  |  |  |  |  |  |  |
|  |  |  |  |  |  |  |

Table 2 Secondary indicators correspondence table

Note：Importance score：Very important =5 points；More important = 4 points；General =3 points；Not very important = 2 points；Not important = 1 point

| Primary indicators | Secondary indicators | Importance score | | | | | Expert opinions |
| --- | --- | --- | --- | --- | --- | --- | --- |
|  |  | 5 | 4 | 3 | 2 | 1 | Modify or delete comments |
| 1.Infectious disease knowledge system | 1.1Basic knowledge of infectious diseases |  |  |  |  |  |  |
|  | 1.2Related knowledge of infectious diseases |  |  |  |  |  |  |
|  | If you have suggested additional items, please fill in the blank lines below (note: please judge their importance) |  |  |  |  |  |  |
|  |  |  |  |  |  |  |  |
|  |  |  |  |  |  |  |  |
| 2.Infectious disease nursing skills | 2.1Protection skills of infectious diseases |  |  |  |  |  |  |
|  | 2.2Critical care skills |  |  |  |  |  |  |
|  | 2.3Airway support skills |  |  |  |  |  |  |
|  | 2.4Basic operational skills under infectious disease protection |  |  |  |  |  |  |
|  | If you have suggested additional items, please fill in the blank lines below (note: please judge their importance) |  |  |  |  |  |  |
|  |  |  |  |  |  |  |  |
|  |  |  |  |  |  |  |  |
| 3.Infectious disease response capacities | 3.1Abilities to interact in an infectious disease outbreak |  |  |  |  |  |  |
|  | 3.2Psychological crisis intervention capacities |  |  |  |  |  |  |
|  | 3.3Critical incident response capabilities |  |  |  |  |  |  |
|  | 3.4Comprehensive rescue capabilities |  |  |  |  |  |  |
|  | If you have suggested additional items, please fill in the blank lines below (note: please judge their importance) |  |  |  |  |  |  |
|  |  |  |  |  |  |  |  |
|  |  |  |  |  |  |  |  |
| 4.Personal Traits | 4.1Ideology and morality |  |  |  |  |  |  |
|  | 4.2Comprehensive quality |  |  |  |  |  |  |
|  | If you have suggested additional items, please fill in the blank lines below (note: please judge their importance) |  |  |  |  |  |  |
|  |  |  |  |  |  |  |  |
|  |  |  |  |  |  |  |  |

Table 3 Tertiary indicators correspondence table

Note：Importance score：Very important =5 points；More important = 4 points；General =3 points；Not very important = 2 points；Not important = 1 point

| Primary indicators | Secondary indicators | Tertiary indicators | Definition of the tertiary indicators | Importance score | | | | | Expert opinions |
| --- | --- | --- | --- | --- | --- | --- | --- | --- | --- |
|  |  |  |  | 5 | 4 | 3 | 2 | 1 | Modify or delete comments |
| 1.Infectious disease knowledge system | 1.1Basic knowledge of infectious diseases | 1.1.1The concept and types of infectious diseases | Understand the concept and classification of different infectious diseases |  |  |  |  |  |  |
|  |  | 1.1.2Pathogenesis of infectious diseasess | Understand the development of different infectious diseases, the mechanisms of tissue damage and pathophysiological changes |  |  |  |  |  |  |
|  |  | 1.1.3Epidemiological characteristics of infectious diseases | Understand the epidemic, seasonal, local and distribution characteristics of different infectious diseases in different populations |  |  |  |  |  |  |
|  |  | 1.1.4Clinical manifestations of infectious diseases | Understand the symptoms and signs of different infectious diseases |  |  |  |  |  |  |
|  |  | 1.1.5Transmission routes of infectious diseases | Understand the modes of transmission of different infectious diseases, such as respiratory transmission, gastrointestinal transmission, and contact transmission. |  |  |  |  |  |  |
|  |  | 1.1.6 Preventive measures for infectious diseases | Learn the prevention methods and measures for different infectious diseases. |  |  |  |  |  |  |
|  |  | 1.1.7Diagnostic criteria for infectious diseases | Understand the diagnostic criteria for patients and suspected patients with different infectious diseases. |  |  |  |  |  |  |
|  |  | 1.1.8Treatment and care of infectious diseases | Understand the principles of treatment and key points of care for different infectious diseases |  |  |  |  |  |  |
|  |  | 1.1.9Reporting process for infectious diseases | Understand the reporting process for different infectious diseases and the completion of infectious disease report cards |  |  |  |  |  |  |
|  |  | 1.1.10Related tests for infectious diseases | Understand the detection methods of different infectious diseases |  |  |  |  |  |  |
|  |  | If you have suggested additional items, please fill in the blank lines below (note: please judge their importance and give a definition of the three levels of indicators). |  |  |  |  |  |  |  |
|  |  |  |  |  |  |  |  |  |  |
|  |  |  |  |  |  |  |  |  |  |
|  | 1.2Related knowledge of infectious diseases | 1.2.1Legal and ethical knowledge | Understand the laws and regulations related to infectious diseases, such as the Law of the People's Republic of China on the Prevention and Control of Infectious Diseases. Protecting the rights and privacy of people with infectious diseases |  |  |  |  |  |  |
|  |  | 1.2.2Informatics knowledge | Master the relevant knowledge of the medical system, telemedicine system, and infectious disease information monitoring systems in the isolation ward, and be skilled in the application. |  |  |  |  |  |  |
|  |  | 1.2.3Foreign language knowledge | Can read information in foreign languages on medical equipment and protective materials and communicate with people in foreign languages. |  |  |  |  |  |  |
|  |  | 1.2.4Work experience | Work experience in infectious diseases, acute and critical diseases, and respiratory departments. |  |  |  |  |  |  |
|  |  | If you have suggested additional items, please fill in the blank lines below (note: please judge their importance and give a definition of the three levels of indicators). |  |  |  |  |  |  |  |
|  |  |  |  |  |  |  |  |  |  |
|  |  |  |  |  |  |  |  |  |  |
| 2.Infectious disease nursing techniques | 2.1Protection skills of infectious diseases | 2.1.1Skills for putting on and taking off protective equipment | Can correctly wear and take off protective clothing, protective masks, goggles and other equipment. |  |  |  |  |  |  |
|  |  | 2.1.2Hand hygiene | Can properly perform hand washing, hygienic hand disinfection, and surgical hand disinfection. |  |  |  |  |  |  |
|  |  | 2.1.3Disinfection and sterilization skills | Understand physical and chemical disinfection and sterilization methods, as well as chemical disinfectant use principles. |  |  |  |  |  |  |
|  |  | If you have suggested additional items, please fill in the blank lines below (note: please judge their importance and give a definition of the three levels of indicators). |  |  |  |  |  |  |  |
|  |  |  |  |  |  |  |  |  |  |
|  |  |  |  |  |  |  |  |  |  |
|  | 2.2Critical care skills | 2.2.1Cardiopulmonary cerebral resuscitation skills | Can correctly perform chest compressions and assist breathing for patients with infectious diseases in cardiac arrest. |  |  |  |  |  |  |
|  |  | 2.2.2Hemodialysis machine use and monitoring skills | Can correctly connect the hemodialysis device, handle the machine alarm, and master the normal value and clinical significance of each index. |  |  |  |  |  |  |
|  |  | 2.2.3ECG monitor using and monitoring skills | Understand the normal values and clinical significance of the ECG monitor indicators and identify common arrhythmias. |  |  |  |  |  |  |
|  |  | 2.2.4Nutrition support skills | Can correctly provide intestinal and external nutrition support for patients with infectious diseases and master their indications and contraindications. |  |  |  |  |  |  |
|  |  | 2.2.5 Hemodynamic monitoring skills | Monitor blood pressure, central venous pressure, pulmonary artery pressure, pulmonary capillary wedge pressure, and cardiac output in patients with infectious diseases using the correct equipment and understand the normal values and clinical significance of each indicator. |  |  |  |  |  |  |
|  |  | 2.2.6 Continuous renal replacement therapy skills | Can correctly implement CRRT for infectious disease patients, prevent the occurrence of complications, master its indications and contraindications, and determine the normal value and clinical significance of each index. |  |  |  |  |  |  |
|  |  | 2.2.7Ventilator using and monitoring skills | Be able to use the ventilator correctly, connect the ventilator tubing and deal with machine alarms. Know the indications and contraindications for different modes of ventilation, the normal values, and clinical significance of each indicator. |  |  |  |  |  |  |
|  |  | 2.2.8 Extracorporeal membrane oxygenation using and monitoring skills | Can correctly assist doctors to conduct ECMO for infectious disease patients, handle machine alarm, master its indications and contraindications, the normal value and clinical significance of each index. |  |  |  |  |  |  |
|  |  | 2.2.9 High-flow oxygen intake device using and monitoring skills | To be able to use high-flow oxygenation devices correctly and deal with machine alarms. Know their indications and contraindications, normal values of indicators and clinical significance. |  |  |  |  |  |  |
|  |  | 2.2.10Defibrillator using skills | To be able to use the defibrillator correctly and understand its indications and contraindications, the normal values of the indicators and their clinical significance. |  |  |  |  |  |  |
|  |  | 2.2.11Prone position ventilation skills | To be able to correctly assist patients with infectious diseases to ventilate in the prone position and to know the indications and contraindications as well as the key points of care. |  |  |  |  |  |  |
|  |  | 2.2.12Electrocardiography machine using and monitoring skills | Proficiency in the use of ECG machines and analysis of ECG results. |  |  |  |  |  |  |
|  |  | 2.2.13Micro pump/syringe pump/infusion pump using skills | Able to use a micropump/syringe pump/infusion pump correctly and deal with machine alarms. |  |  |  |  |  |  |
|  |  | If you have suggested additional items, please fill in the blank lines below (note: please judge their importance and give a definition of the three levels of indicators). |  |  |  |  |  |  |  |
|  |  |  |  |  |  |  |  |  |  |
|  |  |  |  |  |  |  |  |  |  |
|  | 2.3Airway support skills | 2.3.1Cricothyroid membrane puncture skills | Can correctly assist doctors in performing cricothyroid punctures on patients with infectious diseases to prevent complications. |  |  |  |  |  |  |
|  |  | 2.3.2Tracheal intubations/tracheostomy | Able to properly assist doctors in performing tracheal intubation/incision in patients with infectious diseases to prevent complications. |  |  |  |  |  |  |
|  |  | 2.3.3Simple respirator using skills | Can use an improvised breathing apparatus correctly to assist patients with infectious diseases to breathe. |  |  |  |  |  |  |
|  |  | 2.3.4Closed suction skills | Can properly suction patients with infectious diseases who are tracheally intubated/incised to prevent complications. |  |  |  |  |  |  |
|  |  | If you have suggested additional items, please fill in the blank lines below (note: please judge their importance and give a definition of the three levels of indicators). |  |  |  |  |  |  |  |
|  |  |  |  |  |  |  |  |  |  |
|  |  |  |  |  |  |  |  |  |  |
|  | 2.4Basic operational skills under infectious disease protection | 2.4.1Specimen collection, preservation and transportation skills | Be able to collect sputum, blood, and throat swabs from patients with infectious diseases correctly, and know how to preserve and transport various specimens. |  |  |  |  |  |  |
|  |  | 2.4.2Blood gas analysis skills | Be able to use the blood gas analysis machine correctly and understand the normal values and clinical significance of the indicators of blood gas analysis results. |  |  |  |  |  |  |
|  |  | 2.4.3Arteriovenous puncture skills | Can accurately assess the vascular conditions of patients with infectious diseases and perform peripheral and central arterial punctures. |  |  |  |  |  |  |
|  |  | If you have suggested additional items, please fill in the blank lines below (note: please judge their importance and give a definition of the three levels of indicators). |  |  |  |  |  |  |  |
|  |  |  |  |  |  |  |  |  |  |
|  |  |  |  |  |  |  |  |  |  |
| 3.Infectious disease response capacities | 3.1Communication ability in the face of infectious diseases | 3.1.1Communication and coordination abilities | Can communicate smoothly with colleagues and patients with infectious diseases and coordinate the relationship between medical care, doctors and patients, nurses and patients. |  |  |  |  |  |  |
|  |  | 3.1.2Teamwork abilities | Able to work as a team and help each other to care for patients with infectious diseases. |  |  |  |  |  |  |
|  |  | 3.1.3Organization and management abilities | To be able to manage patients with infectious diseases, isolation areas and various materials, and organize patients to participate in rehabilitation training. |  |  |  |  |  |  |
|  |  | If you have suggested additional items, please fill in the blank lines below (note: please judge their importance and give a definition of the three levels of indicators). |  |  |  |  |  |  |  |
|  |  |  |  |  |  |  |  |  |  |
|  |  |  |  |  |  |  |  |  |  |
|  | 3.2Psychological crisis intervention abilities | 3.2.1Psychological risk identification abilities | Mastering the common psychological assessment scale can help identify the psychological changes of patients with infectious diseases. |  |  |  |  |  |  |
|  |  | 3.2.2Psychological care abilities | Be able to use psychological knowledge to counsel patients with infectious diseases and help them recover a healthy psychological state. |  |  |  |  |  |  |
|  |  | 3.2.3Humanistic care | Can respect and care for patients with infectious diseases, and patient-centered. |  |  |  |  |  |  |
|  |  | If you have suggested additional items, please fill in the blank lines below (note: please judge their importance and give a definition of the three levels of indicators). |  |  |  |  |  |  |  |
|  |  |  |  |  |  |  |  |  |  |
|  |  |  |  |  |  |  |  |  |  |
|  | 3.3Critical incident response capabilities | 3.3.1Needlestick injuries emergency treatment abilities | Able to treat wounds correctly, report to the relevant authorities and seek medical help. |  |  |  |  |  |  |
|  |  | 3.3.2Blood/body fluid exposure emergency response abilities | Able to handle exposed blood/body fluids correctly and handle them in a timely manner. |  |  |  |  |  |  |
|  |  | 3.3.3Emergency response to power outages | Ability to deal calmly with power outages and activate backup power in a timely manner. |  |  |  |  |  |  |
|  |  | 3.3.4Fire emergency response | The ability to evacuate infectious diseases patients in a timely and safe manner in the event of a fire to minimise damage and impact |  |  |  |  |  |  |
|  |  | 3.3.5Suicide emergency response | Capable of persuading and rescuing infectious disease patients in a timely manner, as well as reporting to superiors. |  |  |  |  |  |  |
|  |  | 3.3.6Emergency response abilities of insufficient/stopped central oxygen supply | In the event of a shortage or shutdown of central oxygen supply, the ability to continue to supply oxygen to patients with infectious diseases in a timely manner using back-up oxygen, e.g., oxygen cylinders. |  |  |  |  |  |  |
|  |  | 3.3.7Unexplained fainting emergency treatment abilities | Be able to take timely, safe, and effective treatment and care measures when fainting is detected in patients with infectious diseases, health care workers, etc. |  |  |  |  |  |  |
|  |  | 3.3.8Emergency response to protective clothing rupture | Ability to disinfect immediately in the event of a rupture of protective clothing and to take preventive treatment. |  |  |  |  |  |  |
|  |  | 3.3.9Material shortage emergency response abilities | The ability to save supplies and find alternative items when protective supplies are in short supply. |  |  |  |  |  |  |
|  |  | If you have suggested additional items, please fill in the blank lines below (note: please judge their importance and give a definition of the three levels of indicators). |  |  |  |  |  |  |  |
|  |  |  |  |  |  |  |  |  |  |
|  |  |  |  |  |  |  |  |  |  |
|  | 3.4Comprehensive rescue capabilities | 3.4.1Critical thinking capabilities | Be able to question, analyse, reason and judge when working in isolation wards. |  |  |  |  |  |  |
|  |  | 3.4.2Scientific research capacities | Capability to conduct literature reviews, research design, write papers, and data analysis. |  |  |  |  |  |  |
|  |  | 3.4.3Condition observation and disposal abilities | Be able to closely observe changes in the condition of patients with infectious diseases, such as vital signs and consciousness, and take timely action. |  |  |  |  |  |  |
|  |  | 3.4.4Self-directed learning abilities | Be able to take the initiative to use all resources to learn about the treatment, care, and prevention of different infectious diseases. |  |  |  |  |  |  |
|  |  | 3.4.5Injury detection and triage capabilities | Be able to assess and classify patients with infectious diseases or suspected patients. |  |  |  |  |  |  |
|  |  | 3.4.6Clerical writing abilities | Be able to write accurate and error-free records of care for patients with infectious diseases using paper records or electronic systems. |  |  |  |  |  |  |
|  |  | 3.4.7Teaching abilities | To be able to guide colleagues to learn knowledge and techniques related to infectious diseases. To teach rehabilitation training to patients with infectious diseases and provide health education to patients with infectious diseases, etc. |  |  |  |  |  |  |
|  |  | If you have suggested additional items, please fill in the blank lines below (note: please judge their importance and give a definition of the three levels of indicators). |  |  |  |  |  |  |  |
|  |  |  |  |  |  |  |  |  |  |
|  |  |  |  |  |  |  |  |  |  |
| 4.Personal Traits | 4.1Ideology and morality | 4.1.1Unselfish dedication | The ability to sacrifice one's own life and dedicate oneself quietly to infectious disease patients without expecting anything in return. |  |  |  |  |  |  |
|  |  | 4.1.2Spirit of prudence | Be able to act ethically and consciously when unsupervised in isolation wards. |  |  |  |  |  |  |
|  |  | 4.1.3Hardworking spirit | Not be afraid of difficult conditions or tiredness when working in isolation wards. |  |  |  |  |  |  |
|  |  | If you have suggested additional items, please fill in the blank lines below (note: please judge their importance and give a definition of the three levels of indicators). |  |  |  |  |  |  |  |
|  |  |  |  |  |  |  |  |  |  |
|  |  |  |  |  |  |  |  |  |  |
|  | 4.2Comprehensive quality | 4.2.1Physical quality | Ability to carry out nursing tasks while wearing heavy protective gear. |  |  |  |  |  |  |
|  |  | 4.2.2Stress coping abilities | Self-regulation to cope with the high intensity, challenge and contagiousness of work in isolation wards. |  |  |  |  |  |  |
|  |  | 4.2.3Responsibility | Be able to take responsibility for the care of patients with infectious diseases or suspected patients and fulfil the duties of a nurse. |  |  |  |  |  |  |
|  |  | 4.2.4 Self-confidence | Believing that you can solve the challenges you face in isolation wards. |  |  |  |  |  |  |
|  |  | 4.2.5Optimism | Maintain a positive attitude at all times and mobilize positive emotions in patients with infectious diseases and colleagues. |  |  |  |  |  |  |
|  |  | If you have suggested additional items, please fill in the blank lines below (note: please judge their importance and give a definition of the three levels of indicators). |  |  |  |  |  |  |  |
|  |  |  |  |  |  |  |  |  |  |
|  |  |  |  |  |  |  |  |  |  |

Table 4 Please select the basis for your judgement, level of influence and familiarity with the above entry “√” the appropriate column

| Basis of judgement | Level of influence | | | | |
| --- | --- | --- | --- | --- | --- |
|  | large | middle | | small | |
| Theoretical analysis |  |  | |  | |
| Practical experience |  |  | |  | |
| References |  |  | |  | |
| Intuitive aspects |  |  | |  | |
| How familiar are you with the content of this survey | | | | | |
| Degree of familiarity | Very familiar | More familiar | General familiar | Not really familiar | Completely unfamiliar |
|  |  |  |  |  |  |

End of form. Thank you again for your support and help with this subject.

I wish you a happy life. Good luck with your work.
