# Supplementary material for: Construction of a competency evaluation index system for front-line nurses during the outbreak of major infectious diseases: A Delphi study
Source: PLoS One. 2022 Jul 1;17(7):e0270902. doi: 10.1371/journal.pone.0270902 (PMC9249240; doi:10.1371/journal.pone.0270902)
Supplement: S3 File — (DOCX) [file pone.0270902.s003.docx]

**Construction of a competency evaluation index system for front-line nurses during the outbreak of major infectious diseases（the second round）**

Dear Experts:

First of all, I would like to thank you sincerely for your support and help in this study and for your valuable comments in the first round of correspondence.

After the first round of correspondence with 27 experts, members of the subject group, based on a full understanding of the experts' opinions and suggestions, combined with the results of literature review and consultation with medical and nursing staff who had undertaken COVID-2019 or other infectious disease prevention work, conducted active and serious discussions, adjusting and revising the indicators at all levels to form the second round of questionnaires. In order to provide you with a better understanding of the previous round of correspondence from experts and the changes, additions, or additions to the indicators, we have added a description of the changes to the first round of entries in the questionnaire. The second round of consultation is aimed at coordinating expert opinions and determining the weighting of indicators. We would like to invite your valuable comments on the revised indicators again, which will be of great significance to us in finalising the evaluation indicator system for the competence of frontline nursing staff in the event of an outbreak of a major infectious disease. Due to the time-sensitive nature of research and the progress of the study, we kindly ask you to respond to comments and suggestions within a week. If you have any questions about the questionnaire, please do not hesitate to contact us.

Thank you from the bottom of my heart for your support and guidance. I wish you well in your work and good health.

The Second Affiliated Hospital of Chongqing Medical University

Tutor：Xiuni Gan

Postgraduate：Xue Bai

Contacts：Xue Bai Phone / Wechat：15761602836 E-mail：584454151@qq.com

Part 1**：**Front-line nursing staff competence assessment indicators in the outbreak of major infectious disease questionnaire

Instructions for completing the form：

一、This section contains a total of 3 correspondence forms, of which：

Table 1: Primary indicators correspondence table；

Table 2: Secondary indicators correspondence table；

Table 3: Tertiary indicators correspondence table；

二、Please give your rating for the importance of each indicator based on your own experience and knowledge.

1. Importance score：Very important =5 points；More important = 4 points；General =3 points；Not very important = 2 points；Not important = 1 point, Please make a judgement on the relative importance of the indicator and tick the appropriate box.

2. If you think that the indicator description is inaccurate or should be deleted, please fill in the "Comments for amendment or deletion" column or indicate "Delete". If you think that there are additional indicators that we have not considered, please add them in the "Suggested additions" field and rate their importance.

Table 1 Primary indicators correspondence table

Note：Importance score：Very important =5 points；More important = 4 points；General =3 points；Not very important = 2 points；Not important = 1 point

| Primary indicators | Second round importance score | | | | | Expert opinions | |
| --- | --- | --- | --- | --- | --- | --- | --- |
|  | 5 | 4 | 3 | 2 | 1 | Original first-round submission | Modify or delete comments |
| 1.Infectious disease knowledge system |  |  |  |  |  |  |  |
| 2.Related nursing skills for infectious diseases |  |  |  |  |  | Originally：Infectious disease nursing skills |  |
| 3.Related professional abilities for infectious diseases |  |  |  |  |  | Originally：Infectious disease response capacities |  |
| 4.Comprehensive quality |  |  |  |  |  | Originally：Personal Traits |  |
| If you have suggested additional items, please fill in the blank lines below (note: please judge their importance) |  |  |  |  |  |  |  |
|  |  |  |  |  |  |  |  |
|  |  |  |  |  |  |  |  |

Table 2 Secondary indicators correspondence table

Note：Importance score：Very important =5 points；More important = 4 points；General =3 points；Not very important = 2 points；Not important = 1 point

| Primary indicators | Secondary indicators | Second round importance score | | | | | Expert opinions | |
| --- | --- | --- | --- | --- | --- | --- | --- | --- |
|  |  | 5 | 4 | 3 | 2 | 1 | Original first-round submission | Modify or delete comments |
| 1.Infectious disease knowledge system | 1.1Basic knowledge of infectious diseases |  |  |  |  |  |  |  |
|  | 1.2Related knowledge of infectious diseases |  |  |  |  |  |  |  |
|  | If you have suggested additional items, please fill in the blank lines below (note: please judge their importance) |  |  |  |  |  |  |  |
|  |  |  |  |  |  |  |  |  |
|  |  |  |  |  |  |  |  |  |
| 2.Related nursing skills for infectious diseases | 2.1Protection skills of infectious diseases |  |  |  |  |  |  |  |
|  | 2.2Critical care skills |  |  |  |  |  |  |  |
|  | 2.3Related basic nursing skills |  |  |  |  |  | Originally：Basic operational skills under infectious disease protection |  |
|  | If you have suggested additional items, please fill in the blank lines below (note: please judge their importance) |  |  |  |  |  |  |  |
|  |  |  |  |  |  |  |  |  |
|  |  |  |  |  |  |  |  |  |
| 3.Related professional abilities for infectious diseases | 3.1Psychological crisis intervention capacities |  |  |  |  |  |  |  |
|  | 3.2Emergency response abilities |  |  |  |  |  | Originally：Critical incident response capabilities |  |
|  | 3.3Basic rescue capabilities |  |  |  |  |  | Originally：Comprehensive rescue capabilities |  |
|  | If you have suggested additional items, please fill in the blank lines below (note: please judge their importance) |  |  |  |  |  |  |  |
|  |  |  |  |  |  |  |  |  |
|  |  |  |  |  |  |  |  |  |
| 4.Comprehensive quality | 4.1Ideology and morality |  |  |  |  |  |  |  |
|  | 4.2Physical and mental qualities |  |  |  |  |  | Originally：Comprehensive quality |  |
|  | If you have suggested additional items, please fill in the blank lines below (note: please judge their importance) |  |  |  |  |  |  |  |
|  |  |  |  |  |  |  |  |  |
|  |  |  |  |  |  |  |  |  |

Table 3 Tertiary indicators correspondence table

Note：Importance score：Very important =5 points；More important = 4 points；General =3 points；Not very important = 2 points；Not important = 1 point

| Primary indicators | Secondary indicators | Tertiary indicators | Definition of the tertiary indicators | Importance score | | | | | Expert opinions | |
| --- | --- | --- | --- | --- | --- | --- | --- | --- | --- | --- |
|  |  |  |  | 5 | 4 | 3 | 2 | 1 | Original first-round submission | Modify or delete comments |
| 1.Infectious disease knowledge system | 1.1Basic knowledge of infectious diseases | 1.1.1 The concept and types of infectious diseases | Master the concept and classification of different infectious diseases. |  |  |  |  |  |  |  |
|  |  | 1.1.2Pathogenesis of infectious diseasess | Understand the development of different infectious diseases, the mechanisms of tissue damage and pathophysiological changes. |  |  |  |  |  |  |  |
|  |  | 1.1.3Epidemiological characteristics of infectious diseases | Understand the epidemic, seasonal, local and distribution characteristics of different infectious diseases in different populations. |  |  |  |  |  |  |  |
|  |  | 1.1.4Clinical manifestations of infectious diseases | Understand the symptoms and signs of different infectious diseases. |  |  |  |  |  |  |  |
|  |  | 1.1.5Transmission routes of infectious diseases | Understand the modes of transmission of different infectious diseases, such as respiratory transmission, gastrointestinal transmission, and contact transmission. |  |  |  |  |  |  |  |
|  |  | 1.1.6Preventive measures for infectious diseases | Learn the prevention methods and measures for different infectious diseases. |  |  |  |  |  |  |  |
|  |  | 1.1.7Diagnostic criteria for infectious diseases | Understand the diagnostic criteria for patients and suspected patients with different infectious diseases. |  |  |  |  |  |  |  |
|  |  | 1.1.8Treatment and care of infectious diseases | Understand the principles of treatment and key points of care for different infectious diseases. |  |  |  |  |  |  |  |
|  |  | 1.1.9Reporting time and reporting process of infectious diseases | Understand the time limits and reporting process for different infectious diseases. |  |  |  |  |  | Originally:Reporting process for infectious diseases:Understand the reporting process for different infectious diseases and the completion of infectious disease report cards |  |
|  |  | 1.1.10Related tests for infectious diseases | Be familiar with the detection methods for different infectious diseases. |  |  |  |  |  | Originally:Understand the detection methods of different infectious diseases |  |
|  |  | 1.1.11Emergency procedures for infectious diseases | Understand the emergency procedures for different infectious diseases. |  |  |  |  |  | Add |  |
|  |  | 1.1.12Etiology of infectious diseases | Understanding the causes of different infectious diseases. |  |  |  |  |  | Add |  |
|  |  | If you have suggested additional items, please fill in the blank lines below (note: please judge their importance) |  |  |  |  |  |  |  |  |
|  |  |  |  |  |  |  |  |  |  |  |
|  |  |  |  |  |  |  |  |  |  |  |
|  | 1.2Related knowledge of infectious diseases | 1.2.1Legal and ethical knowledge | Understand the laws and regulations related to infectious diseases, such as the Law of the People's Republic of China on the Prevention and Control of Infectious Diseases. Protecting the rights and privacy of people with infectious diseases. |  |  |  |  |  |  |  |
|  |  | 1.2.2Informatics knowledge | Master the relevant knowledge of the medical system, telemedicine system, and infectious disease information monitoring systems in the isolation ward, and be skilled in the application. |  |  |  |  |  |  |  |
|  |  | 1.2.3Knowledge of complex case care for infectious diseases combined with chronic diseases | Knowledge and skills in caring for complex infectious disease cases with comorbid chronic conditions such as hypertension, coronary heart disease, and diabetes. |  |  |  |  |  | Add |  |
|  |  | If you have suggested additional items, please fill in the blank lines below (note: please judge their importance) |  |  |  |  |  |  |  |  |
|  |  |  |  |  |  |  |  |  |  |  |
| 2.Related nursing skills for infectious diseases | 2.1Protection skills of infectious diseases | 2.1.1Skills for putting on and taking off protective equipment | Can correctly wear and take off protective clothing, protective masks, goggles and other equipment. |  |  |  |  |  |  |  |
|  |  | 2.1.2Hand hygiene | Can perform hand hygiene correctly and in accordance with regulations. |  |  |  |  |  | Originally:Can properly perform hand washing, hygienic hand disinfection, and surgical hand disinfection. |  |
|  |  | 2.1.3Disinfection and sterilization skills | Master the disinfection and sterilization methods of the pathogens of various common infectious diseases. |  |  |  |  |  | Originally:Understand physical and chemical disinfection and sterilization methods, as well as chemical disinfectant use principles. |  |
|  |  | 2.1.4Medical waste treatment skills for infectious diseases | Master the treatment methods and procedures of medical waste for different infectious diseases. |  |  |  |  |  | Add |  |
|  |  | If you have suggested additional items, please fill in the blank lines below (note: please judge their importance) |  |  |  |  |  |  |  |  |
|  |  |  |  |  |  |  |  |  |  |  |
|  |  |  |  |  |  |  |  |  |  |  |
|  | 2.2Critical care skills | 2.2.1Cardiopulmonary cerebral resuscitation skills | Can correctly perform chest compressions and assist breathing for patients with infectious diseases in cardiac arrest. |  |  |  |  |  |  |  |
|  |  | 2.2.2ECG monitor using and monitoring skills | Understand the normal values and clinical significance of the ECG monitor indicators and identify common arrhythmias. |  |  |  |  |  |  |  |
|  |  | 2.2.3Nutrition support skills | Be able to correctly administer parenteral and parenteral nutritional support to patients with infectious diseases under the guidance of a doctor, and understand the indications and contraindications. |  |  |  |  |  | Originally:Can correctly provide intestinal and external nutrition support for patients with infectious diseases and master their indications and contraindications. |  |
|  |  | 2.2.4Hemodynamic monitoring skills | Monitor blood pressure, central venous pressure, pulmonary artery pressure, pulmonary capillary wedge pressure, and cardiac output in patients with infectious diseases using the correct equipment and understand the normal values and clinical significance of each indicator. |  |  |  |  |  |  |  |
|  |  | 2.2.5 Continuous renal replacement therapy skills | Can correctly implement CRRT for infectious disease patients, prevent the occurrence of complications, master its indications and contraindications, and determine the normal value and clinical significance of each index. |  |  |  |  |  |  |  |
|  |  | 2.2.6Ventilator using and monitoring skills | Be able to use the ventilator correctly, connect the ventilator tubing and deal with machine alarms. Know the indications and contraindications for different modes of ventilation, the normal values, and clinical significance of each indicator. |  |  |  |  |  |  |  |
|  |  | 2.2.7 Extracorporeal membrane oxygenation using and monitoring skills | Can correctly assist doctors to conduct ECMO for infectious disease patients, handle machine alarm, master its indications and contraindications, the normal value and clinical significance of each index. |  |  |  |  |  |  |  |
|  |  | 2.2.8High-flow oxygen intake device using and monitoring skills | To be able to use high-flow oxygenation devices correctly and deal with machine alarms. Know their indications and contraindications, normal values of indicators and clinical significance. |  |  |  |  |  |  |  |
|  |  | 2.2.9Defibrillator using skills | To be able to use the defibrillator correctly and understand its indications and contraindications, the normal values of the indicators and their clinical significance. |  |  |  |  |  |  |  |
|  |  | 2.2.10Prone position ventilation skills | To be able to correctly assist patients with infectious diseases to ventilate in the prone position and to know the indications and contraindications as well as the key points of care. |  |  |  |  |  |  |  |
|  |  | 2.2.11Electrocardiography machine using and monitoring skills | Proficiency in the use of ECG machines and analysis of ECG results. |  |  |  |  |  |  |  |
|  |  | 2.2.12Micro pump/syringe pump/infusion pump using skills | Able to use a micropump/syringe pump/infusion pump correctly and deal with machine alarms. |  |  |  |  |  |  |  |
|  |  | 2.2.13Cricothyroid membrane puncture skills | Can correctly assist doctors in performing cricothyroid punctures on patients with infectious diseases to prevent complications. |  |  |  |  |  |  |  |
|  |  | 2.2.14Tracheal intubations/tracheostomy | Able to properly assist doctors in performing tracheal intubation/incision in patients with infectious diseases to prevent complications. |  |  |  |  |  |  |  |
|  |  | 2.2.15Simple respirator using skills | Can use an improvised breathing apparatus correctly to assist patients with infectious diseases to breathe. |  |  |  |  |  |  |  |
|  |  | 2.2.16Airbag pressure measurement skills | Can correctly use an airbag manometer for airbag pressure measurement and adjust it when appropriate. |  |  |  |  |  | Add |  |
|  |  | 2.2.17Airway clearance skills | Use a physical or mechanical way to act on the air flow to help the trachea and bronchial sputum discharge or induce cough to discharge sputum. |  |  |  |  |  | Add |  |
|  |  | If you have suggested additional items, please fill in the blank lines below (note: please judge their importance) |  |  |  |  |  |  |  |  |
|  |  |  |  |  |  |  |  |  |  |  |
|  |  |  |  |  |  |  |  |  |  |  |
|  | 2.3Related basic care skills | 2.3.1Specimen collection, preservation and transportation skills | Be able to collect sputum, blood, and throat swabs from patients with infectious diseases correctly, and know how to preserve and transport various specimens. |  |  |  |  |  |  |  |
|  |  | 2.3.2Blood gas analysis skills | Be able to use the blood gas analysis machine correctly and understand the normal values and clinical significance of the indicators of blood gas analysis results. |  |  |  |  |  |  |  |
|  |  | 2.3.3Arteriovenous puncture skills | Can accurately assess the vascular conditions of patients with infectious diseases and perform peripheral and central arterial punctures. |  |  |  |  |  |  |  |
|  |  | If you have suggested additional items, please fill in the blank lines below (note: please judge their importance) |  |  |  |  |  |  |  |  |
|  |  |  |  |  |  |  |  |  |  |  |
|  |  |  |  |  |  |  |  |  |  |  |
| 3.Related professional abilities for infectious diseases | 3.1Psychological crisis intervention abilities | 3.1.1Psychological risk identification abilities | Mastering the common psychological assessment scale can help identify the psychological changes of patients with infectious diseases. |  |  |  |  |  |  |  |
|  |  | 3.1.2Psychological care abilities | Be able to use psychological knowledge to counsel patients with infectious diseases and help them recover a healthy psychological state. |  |  |  |  |  |  |  |
|  |  | 3.1.3Humanistic care | Can respect and care for patients with infectious diseases, and patient-centered. |  |  |  |  |  |  |  |
|  |  | If you have suggested additional items, please fill in the blank lines below (note: please judge their importance) |  |  |  |  |  |  |  |  |
|  |  |  |  |  |  |  |  |  |  |  |
|  |  |  |  |  |  |  |  |  |  |  |
|  | 3.2Emergency response abilities | 3.2.1Needlestick injuries emergency treatment abilities | Able to treat wounds correctly, report to the relevant authorities and seek medical help. |  |  |  |  |  |  |  |
|  |  | 3.2.2Blood/body fluid exposure emergency response abilities | Able to handle exposed blood/body fluids correctly and disinfect them in a timely manner. |  |  |  |  |  | Originally: Able to handle exposed blood/body fluids correctly and handle them in a timely manner. |  |
|  |  | 3.2.3Emergency response to power outages | Ability to deal calmly with power outages and activate backup power in a timely manner. |  |  |  |  |  |  |  |
|  |  | 3.2.4Fire emergency response | The ability to evacuate infectious diseases patients in a timely and safe manner in the event of a fire to minimise damage and impact. |  |  |  |  |  |  |  |
|  |  | 3.2.5Suicide emergency response | Capable of persuading and rescuing infectious disease patients in a timely manner, as well as reporting to superiors. |  |  |  |  |  |  |  |
|  |  | 3.2.6Emergency response abilities of insufficient/stopped central oxygen supply | In the event of a shortage or shutdown of central oxygen supply, the ability to continue to supply oxygen to patients with infectious diseases in a timely manner using back-up oxygen, e.g., oxygen cylinders. |  |  |  |  |  |  |  |
|  |  | 3.2.7Unexplained fainting emergency treatment abilities | Be able to take timely, safe, and effective treatment and care measures when fainting is detected in patients with infectious diseases, health care workers, etc. |  |  |  |  |  |  |  |
|  |  | 3.2.8 Protective equipment breakage emergency response abilities | Ability to disinfect immediately when protective equipment is broken and to take preventive treatment. |  |  |  |  |  | Originally:Emergency response to protective clothing rupture: Ability to disinfect immediately in the event of a rupture of protective clothing and to take preventive treatment. |  |
|  |  | 3.2.9Material shortage emergency response abilities | The ability to find replacement items that meet the level of protection when there is a shortage of protective materials. |  |  |  |  |  | Originally：The ability to save supplies and find alternative items when protective supplies are in short supply. |  |
|  |  | If you have suggested additional items, please fill in the blank lines below (note: please judge their importance) |  |  |  |  |  |  |  |  |
|  |  |  |  |  |  |  |  |  |  |  |
|  |  |  |  |  |  |  |  |  |  |  |
|  | 3.3Basic rescue capabilities | 3.3.1Critical thinking capabilities | Be able to question, analyse, reason and judge when working in isolation wards. |  |  |  |  |  |  |  |
|  |  | 3.3.2Scientific research capacities | Capability to conduct literature reviews, research design, write papers, and data analysis. |  |  |  |  |  |  |  |
|  |  | 3.3.3Condition observation and disposal abilities | Be able to closely observe changes in the condition of patients with infectious diseases, such as vital signs and consciousness, and take timely action. |  |  |  |  |  |  |  |
|  |  | 3.3.4Self-directed learning abilities | Be able to take the initiative to use all resources to learn about the treatment, care, and prevention of different infectious diseases. |  |  |  |  |  |  |  |
|  |  | 3.3.5Triage transfer abilities | Be able to assess and classify patients with infectious diseases or suspected patients. |  |  |  |  |  | Originally: Injury detection and triage capabilities |  |
|  |  | 3.3.6Clerical writing abilities | Be able to write accurate and error-free records of care for patients with infectious diseases using paper records or electronic systems. |  |  |  |  |  |  |  |
|  |  | 3.3.7Teaching abilities | To be able to guide colleagues to learn knowledge and techniques related to infectious diseases. To teach rehabilitation training to patients with infectious diseases and provide health education to patients with infectious diseases, etc. |  |  |  |  |  |  |  |
|  |  | 3.3.8Communication and coordination abilities | Can communicate smoothly with colleagues and patients with infectious diseases and coordinate the relationship between medical care, doctors and patients, nurses and patients. |  |  |  |  |  |  |  |
|  |  | 3.3.9Teamwork abilities | Able to work as a team and help each other to care for patients with infectious diseases. |  |  |  |  |  |  |  |
|  |  | 3.3.10Organization and management abilities | Able to manage infectious disease patients, isolate wards and organise patients to participate in rehabilitation training. |  |  |  |  |  | Originally:To be able to manage patients with infectious diseases, isolation areas and various materials, and organize patients to participate in rehabilitation training. |  |
|  |  | 3.3.11Foreign language skills | Can read information in foreign languages on medical equipment and protective materials and communicate with people in foreign languages. |  |  |  |  |  | Originally:Foreign language knowledge |  |
|  |  | 3.3.12Work experience | Work experience in infectious disease units, intensive care units, and respiratory units, as well as involvement in major infectious disease outbreaks. |  |  |  |  |  | Originally: Work experience in infectious diseases, acute and critical diseases, and respiratory departments. |  |
|  |  | If you have suggested additional items, please fill in the blank lines below (note: please judge their importance) |  |  |  |  |  |  |  |  |
|  |  |  |  |  |  |  |  |  |  |  |
|  |  |  |  |  |  |  |  |  |  |  |
| 4.Comprehensive quality | 4.1Ideology and morality | 4.1.1 Spirit of dedication | The ability to sacrifice one's own life and dedicate oneself quietly to infectious disease patients without expecting anything in return. |  |  |  |  |  | Originally:Unselfish dedication |  |
|  |  | 4.1.2Spirit of prudence | Be able to act ethically and consciously when unsupervised in isolation wards. |  |  |  |  |  |  |  |
|  |  | 4.1.3Hardworking spirit | Not be afraid of difficult conditions or tiredness when working in isolation wards. |  |  |  |  |  |  |  |
|  |  | If you have suggested additional items, please fill in the blank lines below (note: please judge their importance) |  |  |  |  |  |  |  |  |
|  |  |  |  |  |  |  |  |  |  |  |
|  |  |  |  |  |  |  |  |  |  |  |
|  | 4.2Physical and mental qualities | 4.2.1 Physical quality | Ability to carry out nursing tasks while wearing heavy protective gear. |  |  |  |  |  |  |  |
|  |  | 4.2.2Stress coping abilities | Self-regulation to cope with the high intensity, challenge and contagiousness of work in isolation wards. |  |  |  |  |  |  |  |
|  |  | 4.2.3Responsibility | Be able to take responsibility for the care of patients with infectious diseases or suspected patients and fulfil the duties of a nurse. |  |  |  |  |  |  |  |
|  |  | 4.2.4Self-confidence | Believing that you can solve the challenges you face in isolation wards. |  |  |  |  |  |  |  |
|  |  | 4.2.5Optimism | Maintain a positive attitude at all times and mobilize positive emotions in patients with infectious diseases and colleagues. |  |  |  |  |  |  |  |
|  |  | If you have suggested additional items, please fill in the blank lines below (note: please judge their importance) |  |  |  |  |  |  |  |  |
|  |  |  |  |  |  |  |  |  |  |  |
|  |  |  |  |  |  |  |  |  |  |  |

Please select the basis for your judgement, level of influence and familiarity with the above entry “√” the appropriate column

| Basis of judgement | Level of influence | | | | |
| --- | --- | --- | --- | --- | --- |
|  | large | middle | | small | |
| Theoretical analysis |  |  | |  | |
| Practical experience |  |  | |  | |
| References |  |  | |  | |
| Intuitive aspects |  |  | |  | |
| How familiar are you with the content of this survey | | | | | |
| Degree of familiarity | Very familiar | More familiar | General familiar | Not really familiar | Completely unfamiliar |
|  |  |  |  |  |  |

End of form. Thank you again for your support and help with this subject.

I wish you a happy life. Good luck with your work.

Part 2 Analytic hierarchy process to determine the weighting of indicators at the primary, secondary, and tertiary levels

Notes:

This study proposes to use Analytic hierarchy process (AHP) to determine the weights of the evaluation indicators at each level, i.e., to compare the importance of the items at each level, to form a judgment matrix of importance, and to obtain the weight values of the indicators at each level of the evaluation system using AHP special analysis software. Instructions for completing:For the importance rating, please follow the scoring in Table 1 and write the appropriate ratio in the space provided according to your own understanding. The "-" is not required.

Table 1 Methodology for determining the importance of indicators

| Relative Importance (Comparator:Compared) | Definition | Description |
| --- | --- | --- |
| 1 | Equally important | Both the "comparator" and the "compared" aspects are equally relevant. |
| 3 | Slightly more important | Compared to the two factors, "comparator" is slightly more important than "compared". |
| 5 | Obviously important | Compared to the two factors, "comparator" is significantly more important than "compared." |
| 7 | Strongly Important | Compared to the two factors, "comparator" is much more important than "compared." |
| 9 | Absolutely important | Compared to the two factors, "comparator" is absolutely more important than "compared." |
| 1/3 | Slightly less important | Compared to the two factors, "comparator" is slightly less important than "compared." |
| 1/5 | Obviously not important | Compared to the two factors, the "comparator" is significantly less important than the "compared." |
| 1/7 | Not at all important | Compared to the two factors, "comparator" is strongly less important than "compared." |
| 1/9 | Absolutely not important | Compared to the two factors, "comparator" is absolutely less important than "compared." |
| 2、4、6、8或 1/2、1/4、1/6、1/8 |  | Indicates the median value between two adjacent scales, e.g., comparator: compared is 4, meaning comparator: comparatee is somewhere in the middle of slightly more important (3) and obviously important (5) in terms of importance. |

一、Comparison of primary indicators

| Compared  Comparator | 1.Infectious disease knowledge system | 2.Related nursing skills for infectious diseases | 3.Related professional abilities for infectious diseases | 4.Comprehensive quality |
| --- | --- | --- | --- | --- |
| 1.Infectious disease knowledge system | － |  |  |  |
| 2.Related nursing skills for infectious diseases | － | － |  |  |
| 3.Related professional abilities for infectious diseases | － | － | － |  |
| 4.Comprehensive quality | － | － | － | － |

二、Comparison of secondary indicators

（1）Comparison of the internal index of the infectious disease knowledge system

| Compared  Comparator | 1.1Basic knowledge of infectious diseases | 1.2Related knowledge of infectious diseases |
| --- | --- | --- |
| 1.1Basic knowledge of infectious diseases | － |  |
| 1.2Related knowledge of infectious diseases | － | － |

（2）Comparison of internal indicators of related nursing skills for infectious diseases

| Compared  Comparator | 2.1Protection skills of infectious diseases | 2.2Critical care skills | 2.3Related basic nursing skills |
| --- | --- | --- | --- |
| 2.1Protection skills of infectious diseases | － |  |  |
| 2.2Critical care skills | － | － |  |
| 2.3Related basic nursing skills | － | － | － |

（3）Comparison of internal indicators of related professional abilities for infectious diseases

| Compared  Comparator | 3.1Psychological crisis intervention abilities | 3.2Emergency response abilities | 3.3Basic rescue capabilities |
| --- | --- | --- | --- |
| 3.1Psychological crisis intervention abilities | － |  |  |
| 3.2Emergency response abilities | － | － |  |
| 3.3Basic rescue capabilities | － | － | － |

（4）Comparison of internal indicators of comprehensive quality

| Compared  Comparator | 4.1Ideology and morality | 4.2Physical and mental qualities |
| --- | --- | --- |
| 4.1Ideology and morality | － |  |
| 4.2Physical and mental qualities | － | － |

三、Comparison of tertiary indicators

（1）Comparison of the internal indicators of the basic knowledge of infectious diseases

| Compared  Comparator | 1.1.1The concept and types of infectious diseases | 1.1.2Pathogenesis of infectious diseasess | 1.1.3Epidemiological characteristics of infectious diseases | 1.1.4Clinical manifestations of infectious diseases | 1.1.5Transmission routes of infectious diseases | 1.1.6Preventive measures for infectious diseases | 1.1.7Diagnostic criteria for infectious diseases | 1.1.8Treatment and care of infectious diseases | 1.1.9Reporting time and reporting process of infectious diseases | 1.1.10Related tests for infectious diseases | 1.1.11Emergency procedures for infectious diseases | 1.1.12Etiology of infectious diseases |
| --- | --- | --- | --- | --- | --- | --- | --- | --- | --- | --- | --- | --- |
| 1.1.1The concept and types of infectious diseases | － |  |  |  |  |  |  |  |  |  |  |  |
| 1.1.2Pathogenesis of infectious diseasess | － | － |  |  |  |  |  |  |  |  |  |  |
| 1.1.3Epidemiological characteristics of infectious diseases | － | － | － |  |  |  |  |  |  |  |  |  |
| 1.1.4Clinical manifestations of infectious diseases | － | － | － | － |  |  |  |  |  |  |  |  |
| 1.1.5Transmission routes of infectious diseases | － | － | － | － | － |  |  |  |  |  |  |  |
| 1.1.6Preventive measures for infectious diseases | － | － | － | － | － | － |  |  |  |  |  |  |
| 1.1.7Diagnostic criteria for infectious diseases | － | － | － | － | － | － | － |  |  |  |  |  |
| 1.1.8Treatment and care of infectious diseases | － | － | － | － | － | － | － | － |  |  |  |  |
| 1.1.9Reporting time and reporting process of infectious diseases | － | － | － | － | － | － | － | － | － |  |  |  |
| 1.1.10Related tests for infectious diseases | － | － | － | － | － | － | － | － | － | － |  |  |
| 1.1.11Emergency procedures for infectious diseases | － | － | － | － | － | － | － | － | － | － | － |  |
| 1.1.12Etiology of infectious diseases | － | － | － | － | － | － | － | － | － | － | － | － |

1. Comparison of internal indicators of related knowledge of infectious diseases

| Compared  Comparator | 1.2.1Legal and ethical knowledge | 1.2.2Informatics knowledge | 1.2.3Knowledge of complex case care for infectious diseases combined with chronic diseases |
| --- | --- | --- | --- |
| 1.2.1Legal and ethical knowledge | － |  |  |
| 1.2.2Informatics knowledge | － | － |  |
| 1.2.3Knowledge of complex case care for infectious diseases combined with chronic diseases | － | － | － |

（3）Comparison of the internal indicators of protection skills of infectious diseases

| Compared  Comparator | 2.1.1Skills for putting on and taking off protective equipment | 2.1.2Hand hygiene | 2.1.3Disinfection and sterilization skills | 2.1.4Medical waste treatment skills for infectious diseases |
| --- | --- | --- | --- | --- |
| 2.1.1Skills for putting on and taking off protective equipment | － |  |  |  |
| 2.1.2Hand hygiene | － | － |  |  |
| 2.1.3Disinfection and sterilization skills | － | － | － |  |
| 2.1.4Medical waste treatment skills for infectious diseases | － | － | － | － |

（4）Comparison of the internal indicators of critical care skills

| Compared  Comparator | 2.2.1Cardiopulmonary cerebral resuscitation skills | 2.2.2ECG monitor using and monitoring skills | 2.2.3Nutrition support skills | 2.2.4Hemodynamic monitoring skills | 2.2.5 Continuous renal replacement therapy skills | 2.2.6Ventilator using and monitoring skills | 2.2.7 Extracorporeal membrane oxygenation using and monitoring skills | 2.2.8High-flow oxygen intake device using and monitoring skills | 2.2.9Defibrillator using skills | 2.2.10Prone position ventilation skills | 2.2.11Electrocardiography machine using and monitoring skills | 2.2.12Micro pump/syringe pump/infusion pump using skills | 2.2.13Cricothyroid membrane puncture skills | 2.2.14Tracheal intubations/tracheostomy | 2.2.15Simple respirator using skills | 2.2.16Airbag pressure measurement skills | 2.2.17Airway clearance skills |
| --- | --- | --- | --- | --- | --- | --- | --- | --- | --- | --- | --- | --- | --- | --- | --- | --- | --- |
| 2.2.1Cardiopulmonary cerebral resuscitation skills | － |  |  |  |  |  |  |  |  |  |  |  |  |  |  |  |  |
| 2.2.2ECG monitor using and monitoring skills | － | － |  |  |  |  |  |  |  |  |  |  |  |  |  |  |  |
| 2.2.3Nutrition support skills | － | － | － |  |  |  |  |  |  |  |  |  |  |  |  |  |  |
| 2.2.4Hemodynamic monitoring skills | － | － | － | － |  |  |  |  |  |  |  |  |  |  |  |  |  |
| 2.2.5 Continuous renal replacement therapy skills | － | － | － | － | － |  |  |  |  |  |  |  |  |  |  |  |  |
| 2.2.6Ventilator using and monitoring skills | － | － | － | － | － | － |  |  |  |  |  |  |  |  |  |  |  |
| 2.2.7 Extracorporeal membrane oxygenation using and monitoring skills | － | － | － | － | － | － | － |  |  |  |  |  |  |  |  |  |  |
| 2.2.8High-flow oxygen intake device using and monitoring skills | － | － | － | － | － | － | － | － |  |  |  |  |  |  |  |  |  |
| 2.2.9Defibrillator using skills | － | － | － | － | － | － | － | － | － |  |  |  |  |  |  |  |  |
| 2.2.10Prone position ventilation skills | － | － | － | － | － | － | － | － | － | － |  |  |  |  |  |  |  |
| 2.2.11Electrocardiography machine using and monitoring skills | － | － | － | － | － | － | － | － | － | － | － |  |  |  |  |  |  |
| 2.2.12Micro pump/syringe pump/infusion pump using skills | － | － | － | － | － | － | － | － | － | － | － | － |  |  |  |  |  |
| 2.2.13Cricothyroid membrane puncture skills | － | － | － | － | － | － | － | － | － | － | － | － | － |  |  |  |  |
| 2.2.14Tracheal intubations/tracheostomy | － | － | － | － | － | － | － | － | － | － | － | － | － | － |  |  |  |
| 2.2.15Simple respirator using skills | － | － | － | － | － | － | － | － | － | － | － | － | － | － | － |  |  |
| 2.2.16Airbag pressure measurement skills | － | － | － | － | － | － | － | － | － | － | － | － | － | － | － | － |  |
| 2.2.17Airway clearance skills | － | － | － | － | － | － | － | － | － | － | － | － | － | － | － | － | － |

（5）Comparison of the internal indicators of related basic nursing skills

| Compared  Comparator | 2.3.1Specimen collection, preservation and transportation skills | 2.3.2Blood gas analysis skills | 2.3.3Arteriovenous puncture skills |
| --- | --- | --- | --- |
| 2.3.1Specimen collection, preservation and transportation skills | － |  |  |
| 2.3.2Blood gas analysis skills | － | － |  |
| 2.3.3Arteriovenous puncture skills | － | － | － |

（6）Comparison of internal indicators of Psychological crisis intervention abilities

| Compared  Comparator | 3.1.1Psychological risk identification abilities | 3.1.2Psychological care abilities | 3.1.3Humanistic care |
| --- | --- | --- | --- |
| 3.1.1Psychological risk identification abilities | － |  |  |
| 3.1.2Psychological care abilities | － | － |  |
| 3.1.3Humanistic care | － | － | － |

（7）Comparison of internal indicators of emergency response abilities

| Compared  Comparator | 3.2.1Needlestick injuries emergency treatment abilities | 3.2.2Blood/body fluid exposure emergency response abilities | 3.2.3Emergency response to power outages | 3.2.4Fire emergency response | 3.2.5Suicide emergency response | 3.2.6Emergency response abilities of insufficient/stopped central oxygen supply | 3.2.7Unexplained fainting emergency treatment abilities | 3.2.8Protective equipment breakage emergency response abilities | 3.2.9Material shortage emergency response abilities |
| --- | --- | --- | --- | --- | --- | --- | --- | --- | --- |
| 3.2.1Needlestick injuries emergency treatment abilities | － |  |  |  |  |  |  |  |  |
| 3.2.2Blood/body fluid exposure emergency response abilities | － | － |  |  |  |  |  |  |  |
| 3.2.3Emergency response to power outages | － | － | － |  |  |  |  |  |  |
| 3.2.4Fire emergency response | － | － | － | － |  |  |  |  |  |
| 3.2.5Suicide emergency response | － | － | － | － | － |  |  |  |  |
| 3.2.6Emergency response abilities of insufficient/stopped central oxygen supply | － | － | － | － | － | － |  |  |  |
| 3.2.7Unexplained fainting emergency treatment abilities | － | － | － | － | － | － | － |  |  |
| 3.2.8Protective equipment breakage emergency response abilities | － | － | － | － | － | － | － | － |  |
| 3.2.9Material shortage emergency response abilities | － | － | － | － | － | － | － | － | － |

(8)Comparison of internal indicators of basic rescue capabilities

| Compared  Comparator | 3.3.1Critical thinking capabilities | 3.3.2Scientific research capacities | 3.3.3Condition observation and disposal abilities | 3.3.4Self-directed learning abilities | 3.3.5Triage transfer abilities | 3.3.6Clerical writing abilities | 3.3.7Teaching abilities | 3.3.8Communication and coordination abilities | 3.3.9Teamwork abilities | 3.3.10Organization and management abilities | 3.3.11Foreign language skills | 3.3.12Work experience |
| --- | --- | --- | --- | --- | --- | --- | --- | --- | --- | --- | --- | --- |
| 3.3.1Critical thinking capabilities | － |  |  |  |  |  |  |  |  |  |  |  |
| 3.3.2Scientific research capacities | － | － |  |  |  |  |  |  |  |  |  |  |
| 3.3.3Condition observation and disposal abilities | － | － | － |  |  |  |  |  |  |  |  |  |
| 3.3.4Self-directed learning abilities | － | － | － | － |  |  |  |  |  |  |  |  |
| 3.3.5Triage transfer abilities | － | － | － | － | － |  |  |  |  |  |  |  |
| 3.3.6Clerical writing abilities | － | － | － | － | － | － |  |  |  |  |  |  |
| 3.3.7Teaching abilities | － | － | － | － | － | － | － |  |  |  |  |  |
| 3.3.8Communication and coordination abilities | － | － | － | － | － | － | － | － |  |  |  |  |
| 3.3.9Teamwork abilities | － | － | － | － | － | － | － | － | － |  |  |  |
| 3.3.10Organization and management abilities | － | － | － | － | － | － | － | － | － | － |  |  |
| 3.3.11Foreign language skills | － | － | － | － | － | － | － | － | － | － | － |  |
| 3.3.12Work experience | － | － | － | － | － | － | － | － | － | － | － | － |

（9）Comparison of internal indicators of ideology and morality

| Compared  Comparator | 4.1.1Spirit of dedication | 4.1.2Spirit of prudence | 4.1.3Hardworking spirit |
| --- | --- | --- | --- |
| 4.1.1Spirit of dedication | － |  |  |
| 4.1.2Spirit of prudence | － | － |  |
| 4.1.3Hardworking spirit | － | － | － |

（10）Comparison of internal indicators of physical and mental qualities

| Compared  Comparator | 4.2.1Physical quality | 4.2.2Stress coping abilities | 4.2.3Responsibility | 4.2.4Self-confidence | 4.2.5Optimism |
| --- | --- | --- | --- | --- | --- |
| 4.2.1Physical quality | － |  |  |  |  |
| 4.2.2Stress coping abilities | － | － |  |  |  |
| 4.2.3Responsibility | － | － | － |  |  |
| 4.2.4Self-confidence | － | － | － | － |  |
| 4.2.5Optimism | － | － | － | － | － |
