# Supplementary material for: Construction of a competency evaluation index system for front-line nurses during the outbreak of major infectious diseases: A Delphi study
Source: PLoS One. 2022 Jul 1;17(7):e0270902. doi: 10.1371/journal.pone.0270902 (PMC9249240; doi:10.1371/journal.pone.0270902)
Supplement: S4 File — (DOCX) [file pone.0270902.s004.docx]

**突发重大传染病疫情下一线护理人员胜任力素质评价指标体系专家函询问卷（第一轮）**

尊敬的专家：

您好，首先衷心感谢您愿意担任本项研究的函询专家！

我是重庆医科大学附属第二医院甘秀妮教授的硕士研究生白雪，我们正在进行《突发重大传染病疫情下一线护理人员胜任力评价模型的开发与信效度检验》。在前期文献研究及质性访谈的基础上，经过我院专家小组会议，初步形成了突发重大传染病疫情下一线护理人员胜任力评价指标体系框架，包括4个一级指标，12个二级指标，67个三级指标，在指标覆盖面、内容重要性方面，仍需各位专家进一步指导。

本次专家函询表包括两个部分，第一部分为函询专家基本信息调查表；第二部分为重大传染病疫情下一线护理人员胜任力评价指标函询表，即表1-4。对于您所提供的信息我们将严格保密，仅用于本次研究中。

鉴于您在本领域较高的学术造诣、承担过COVID-2019或其它传染病防疫工作，具备丰富的临床经验，恳切希望能够得到您的指导和帮助，您的意见将作为我们研究的重要依据！本研究拟进行两轮专家函询，本轮函询拟确定各指标内容，第二轮函询确定权重。由于科研的时效性和研究进度的推进，恳请您在**1周之内**回复意见和建议。如果您对问卷有任何疑问，请随时联系我们。

衷心地感谢您的支持与指导！祝您工作顺利，身体健康！

重庆医科大学附属第二医院

导师：甘秀妮

研究生：白 雪

联系人：白雪 电话/微信：15761602836 邮箱：584454151@qq.com

**第一部分：专家基本信息调查表**

**1.此调查表旨在了解您的情况，资料只用于统计分析，绝对保密，决不它用。请您根据个人实际情况填写，或在合适选项上打“√”或标红。如需进一步说明，请在相应栏中标注。**

| **姓名** |  | **性别** | **□男 □女** | | **年龄** | **____岁** |
| --- | --- | --- | --- | --- | --- | --- |
| **最高学历** | **□博士**  **□硕士**  **□本科**  **□专科**  **□其他：____** | **研究方向** | **□重症医学**  **□急救医学**  **□流行病学**  **□公共卫生**  **□重症护理**  **□急救护理**  **□护理管理**  **□其他：_____** | | **工作年限** | **____年** |
| **职务** | **□院长 □副院长**  **□科主任 □科副主任**  **□护理部主任 □护理部副主任**  **□科护士长 □护士长**  **□带教组长 □其他：_____** | | | | **职称** | **□正高级**  **□副高级**  **□中级**  **□其他：_____** |
| **是否导师** | **□博士生导师 □研究生导师 □否** | | | | | |
| **工作单位** |  | | | | | |
| **联系方式** |  | | | **电子邮箱** | |  |

第二部分：突发重大传染病疫情下一线护理人员胜任力评价指标函询表

填表说明：

**一、**此部分共包含4个函询表，其中：

表1：一级指标函询表；

表2：二级指标函询表；

表3：三级指标函询表；

表4：请选择您对以上条目的判断依据、影响程度及熟悉程度打分。

二、请根据您自己的经验和知识，就每个指标的重要程度给予您的评分。

1.重要性评分：非常重要=5分；比较重要=4分；一般=3分；不太重要=2分；不重要=1分，请您对指标的相对重要程度做出判断，然后在相应栏里打“√”。

2.如果您认为指标描述不准确或应删除，请在“修改或删除意见”栏内填写修改内容或注明“删除”。对于您认为还有我们未考虑到的需要增加的指标，请在“建议增加项目”空白栏内补充，修改和补充的内容请同样判断其重要程度，请勿有空项或漏项。

**表1 一级指标函询表**

**注：重要性评分：非常重要=5分；比较重要=4分；一般=3分；不太重要=2分；不重要=1分**

| **一级指标** | **重要性评分** | | | | | **专家意见** |
| --- | --- | --- | --- | --- | --- | --- |
|  | **5** | **4** | **3** | **2** | **1** | **修改或删除意见** |
| **1.传染病知识体系** |  |  |  |  |  |  |
| **2.传染病护理技术** |  |  |  |  |  |  |
| **3.传染病应对能力** |  |  |  |  |  |  |
| **4.个人特质** |  |  |  |  |  |  |
| **如有建议增加项目，请在下面空行填写（注：请判断其重要程度）** |  |  |  |  |  |  |
|  |  |  |  |  |  |  |
|  |  |  |  |  |  |  |

**表2 二级指标函询表**

**注：重要性评分：非常重要=5分；比较重要=4分；一般=3分；不太重要=2分；不重要=1分**

| **一级指标** | **二级指标** | **重要性评分** | | | | | **专家意见** |
| --- | --- | --- | --- | --- | --- | --- | --- |
|  |  | **5** | **4** | **3** | **2** | **1** | **修改或删除意见** |
| **1.传染病知识体系** | **1.1传染病基础知识** |  |  |  |  |  |  |
|  | **1.2传染病相关知识** |  |  |  |  |  |  |
|  | **如有建议增加项目，请在下面空行填写（注：请判断其重要程度）** |  |  |  |  |  |  |
|  |  |  |  |  |  |  |  |
|  |  |  |  |  |  |  |  |
| **2.传染病护理技术** | **2.1传染病防护技术** |  |  |  |  |  |  |
|  | **2.2重症救护技术** |  |  |  |  |  |  |
|  | **2.3气道支持技术** |  |  |  |  |  |  |
|  | **2.4传染病防护下基础操作技术** |  |  |  |  |  |  |
|  | **如有建议增加项目，请在下面空行填写（注：请判断其重要程度）** |  |  |  |  |  |  |
|  |  |  |  |  |  |  |  |
|  |  |  |  |  |  |  |  |
| **3.传染病应对能力** | **3.1传染病疫情下交往能力** |  |  |  |  |  |  |
|  | **3.2心理危机干预能力** |  |  |  |  |  |  |
|  | **3.3突发事件应对能力** |  |  |  |  |  |  |
|  | **3.4综合救援能力** |  |  |  |  |  |  |
|  | **如有建议增加项目，请在下面空行填写（注：请判断其重要程度）** |  |  |  |  |  |  |
|  |  |  |  |  |  |  |  |
|  |  |  |  |  |  |  |  |
| **4.个人特质** | **4.1思想品质** |  |  |  |  |  |  |
|  | **4.2综合素质** |  |  |  |  |  |  |
|  | **如有建议增加项目，请在下面空行填写（注：请判断其重要程度）** |  |  |  |  |  |  |
|  |  |  |  |  |  |  |  |
|  |  |  |  |  |  |  |  |

**表3 三级指标函询表**

**注：重要性评分：非常重要=5分；比较重要=4分；一般=3分；不太重要=2分；不重要=1分**

| **一级指标** | **二级指标** | **三级指标** | **三级指标定义** | **重要性评分** | | | | | **专家意见** |
| --- | --- | --- | --- | --- | --- | --- | --- | --- | --- |
|  |  |  |  | **5** | **4** | **3** | **2** | **1** | **修改或删除意见** |
| **1.传染病知识体系** | **1.1传染病基础知识** | **1.1.1传染病的概念及类型** | **掌握不同传染病的概念以及分类** |  |  |  |  |  |  |
|  |  | **1.1.2传染病的发病机制** | **掌握不同传染病的发生发展过程、组织损伤机制以及病理生理变化等** |  |  |  |  |  |  |
|  |  | **1.1.3传染病的流行病学特征** | **掌握不同传染病的流行性、季节性、地方性和在不同人群中的分布特点** |  |  |  |  |  |  |
|  |  | **1.1.4传染病的临床表现** | **掌握不同传染病的症状、体征等** |  |  |  |  |  |  |
|  |  | **1.1.5传染病的传播途径** | **掌握不同传染病的传播途径，如呼吸道传播、消化道传播、接触传播等** |  |  |  |  |  |  |
|  |  | **1.1.6传染病的预防措施** | **掌握不同传染病的预防方法及措施** |  |  |  |  |  |  |
|  |  | **1.1.7传染病的诊断标准** | **掌握不同传染病患者、疑似患者的诊断标准** |  |  |  |  |  |  |
|  |  | **1.1.8传染病的治疗及护理** | **掌握不同传染病的治疗原则和护理要点** |  |  |  |  |  |  |
|  |  | **1.1.9传染病的报告流程** | **掌握不同传染病的上报流程、传染病报告卡的填写** |  |  |  |  |  |  |
|  |  | **1.1.10传染病的相关检测** | **掌握不同传染病的检测方法** |  |  |  |  |  |  |
|  |  | **如有建议增加项目，请在下面空行填写（注：请判断其重要程度，并给出三级指标定义）** |  |  |  |  |  |  |  |
|  |  |  |  |  |  |  |  |  |  |
|  |  |  |  |  |  |  |  |  |  |
|  | **1.2传染病相关知识** | **1.2.1法律、伦理知识** | **掌握传染病相关的法律法规，如《中华人民共和国传染病防治法》等，保护传染病患者的权利和隐私** |  |  |  |  |  |  |
|  |  | **1.2.2信息学知识** | **掌握隔离病区医疗系统、远程医学系统、传染病信息监测系统使用的相关知识，并能熟练应用** |  |  |  |  |  |  |
|  |  | **1.2.3外语知识** | **能阅读医疗仪器和防护物资上的外语信息，用外语与人交流** |  |  |  |  |  |  |
|  |  | **1.2.4工作经验** | **具备传染病、急危重症、呼吸科等工作经历** |  |  |  |  |  |  |
|  |  | **如有建议增加项目，请在下面空行填写（注：请判断其重要程度，并给出三级指标定义）** |  |  |  |  |  |  |  |
|  |  |  |  |  |  |  |  |  |  |
|  |  |  |  |  |  |  |  |  |  |
| **2.传染病护理技术** | **2.1传染病防护技术** | **2.1.1防护装备穿脱技术** | **能正确穿脱防护服、防护面罩、护目镜等装备** |  |  |  |  |  |  |
|  |  | **2.1.2手卫生** | **能正确进行洗手、卫生手消毒、外科手消毒** |  |  |  |  |  |  |
|  |  | **2.1.3消毒灭菌技术** | **掌握物理、化学消毒灭菌法，以及化学消毒剂的使用原则** |  |  |  |  |  |  |
|  |  | **如有建议增加项目，请在下面空行填写（注：请判断其重要程度，并给出三级指标定义）** |  |  |  |  |  |  |  |
|  |  |  |  |  |  |  |  |  |  |
|  |  |  |  |  |  |  |  |  |  |
|  | **2.2重症救护技术** | **2.2.1心肺脑复苏技术** | **能正确为心脏骤停的传染病患者进行胸外心脏按压、人工辅助呼吸等** |  |  |  |  |  |  |
|  |  | **2.2.2血透机使用及监测技术** | **能正确连接血液透析装置，处理机器报警，掌握各指标的正常值及临床意义** |  |  |  |  |  |  |
|  |  | **2.2.3心电监护仪使用及监测技术** | **掌握心电监护仪各指标的正常值及临床意义，识别常见心律失常** |  |  |  |  |  |  |
|  |  | **2.2.4营养支持技术** | **能正确为传染病患者进行肠内外营养支持，掌握其适应症和禁忌症** |  |  |  |  |  |  |
|  |  | **2.2.5血流动力学监测技术** | **能正确使用仪器对传染病患者血压，中心静脉压，肺动脉压，肺毛细血管楔压，心输出量等进行监测，掌握各指标的正常值及临床意义** |  |  |  |  |  |  |
|  |  | **2.2.6 CRRT技术** | **能正确为传染病患者实施CRRT，预防并发症发生，掌握其适应症和禁忌症，各指标的正常值及临床意义** |  |  |  |  |  |  |
|  |  | **2.2.7呼吸机使用及监测技术** | **能正确使用呼吸机，连接呼吸机管道，处理机器报警；掌握呼吸机不同模式的适应症和禁忌症，各指标的正常值及临床意义** |  |  |  |  |  |  |
|  |  | **2.2.8 ECMO使用及监测技术** | **能正确协助医生为传染病患者进行ECMO，处理机器报警；掌握其适应症和禁忌症，各指标的正常值及临床意义** |  |  |  |  |  |  |
|  |  | **2.2.9高流量吸氧装置使用及监测技术** | **能正确使用高流量吸氧装置，处理机器报警；掌握其适应症和禁忌症，各指标的正常值及临床意义** |  |  |  |  |  |  |
|  |  | **2.2.10除颤仪使用技术** | **能正确使用除颤仪，掌握其适应症和禁忌症，各指标的正常值及临床意义** |  |  |  |  |  |  |
|  |  | **2.2.11俯卧位通气技术** | **能正确帮助传染病患者进行俯卧位通气，掌握其适应症和禁忌症以及护理要点** |  |  |  |  |  |  |
|  |  | **2.2.12心电图机使用技术** | **能熟练使用心电图机，并对心电图结果进行分析** |  |  |  |  |  |  |
|  |  | **2.2.13微量泵/注射泵/输液泵使用技术** | **能正确使用微量泵/注射泵/输液泵，处理机器报警** |  |  |  |  |  |  |
|  |  | **如有建议增加项目，请在下面空行填写（注：请判断其重要程度，并给出三级指标定义）** |  |  |  |  |  |  |  |
|  |  |  |  |  |  |  |  |  |  |
|  |  |  |  |  |  |  |  |  |  |
|  | **2.3气道支持技术** | **2.3.1环甲膜穿刺术** | **能正确协助医生为传染病患者进行环甲膜穿刺，预防并发症发生** |  |  |  |  |  |  |
|  |  | **2.3.2气管插管/切开技术** | **能正确协助医生为传染病患者实施气管插管/切开，预防并发症发生** |  |  |  |  |  |  |
|  |  | **2.3.3简易呼吸器使用技术** | **能正确使用简易呼吸器辅助传染病患者呼吸** |  |  |  |  |  |  |
|  |  | **2.3.4密闭式吸痰技术** | **能正确为气管插管/切开的传染病患者进行吸痰，预防并发症发生** |  |  |  |  |  |  |
|  |  | **如有建议增加项目，请在下面空行填写（注：请判断其重要程度，并给出三级指标定义）** |  |  |  |  |  |  |  |
|  |  |  |  |  |  |  |  |  |  |
|  |  |  |  |  |  |  |  |  |  |
|  | **2.4传染病防护下基础操作技术** | **2.4.1标本采集、保存及运输技术** | **能正确采集传染病患者的痰、血液、咽拭子等标本，掌握各种标本的保存方法及运输方式** |  |  |  |  |  |  |
|  |  | **2.4.2血气分析技术** | **能正确使用血气分析机，掌握血气分析结果各指标的正常值及临床意义** |  |  |  |  |  |  |
|  |  | **2.4.3动静脉穿刺技术** | **能准确评估传染病患者的血管条件，进行外周和中心动静脉穿刺** |  |  |  |  |  |  |
|  |  | **如有建议增加项目，请在下面空行填写（注：请判断其重要程度，并给出三级指标定义）** |  |  |  |  |  |  |  |
|  |  |  |  |  |  |  |  |  |  |
|  |  |  |  |  |  |  |  |  |  |
| **3.传染病应对能力** | **3.1传染病疫情下交往能力** | **3.1.1沟通协调能力** | **能够与同事、传染病患者沟通顺畅，同时协调好医护、医患及护患关系** |  |  |  |  |  |  |
|  |  | **3.1.2团队协作能力** | **能够发挥团队精神，互帮互助，共同护理好传染病患者** |  |  |  |  |  |  |
|  |  | **3.1.3组织管理能力** | **能够管理好传染病患者、隔离病区以及各种物资，组织患者参加康复训练** |  |  |  |  |  |  |
|  |  | **如有建议增加项目，请在下面空行填写（注：请判断其重要程度，并给出三级指标定义）** |  |  |  |  |  |  |  |
|  |  |  |  |  |  |  |  |  |  |
|  |  |  |  |  |  |  |  |  |  |
|  | **3.2心理危机干预能力** | **3.2.1心理风险识别能力** | **掌握常见的心理评估量表，能够及时识别传染病患者的心理变化** |  |  |  |  |  |  |
|  |  | **3.2.2心理护理能力** | **能够运用心理学知识对传染病患者进行心理疏导，帮助患者恢复健康的心理状态** |  |  |  |  |  |  |
|  |  | **3.2.3人文关怀能力** | **能够尊重、关心传染病患者，以患者为中心** |  |  |  |  |  |  |
|  |  | **如有建议增加项目，请在下面空行填写（注：请判断其重要程度，并给出三级指标定义）** |  |  |  |  |  |  |  |
|  |  |  |  |  |  |  |  |  |  |
|  |  |  |  |  |  |  |  |  |  |
|  | **3.3突发事件应对能力** | **3.3.1针刺伤应急处理** | **能够正确处理伤口，上报相关部门，寻求医疗帮助** |  |  |  |  |  |  |
|  |  | **3.3.2血液/体液暴露应急处理** | **能够正确处理暴露在外的血液/体液，及时进行消毒** |  |  |  |  |  |  |
|  |  | **3.3.3停电应急处理** | **停电时能够冷静处理，及时启动备用电源** |  |  |  |  |  |  |
|  |  | **3.3.4火灾应急处理** | **发生火灾时能够及时、安全撤离传染病患者，把损害和影响降到最低** |  |  |  |  |  |  |
|  |  | **3.3.5自杀应急处理** | **能够及时劝解、抢救传染病患者，并向上级汇报** |  |  |  |  |  |  |
|  |  | **3.3.6中心供氧不足/停氧应急处理** | **中心供氧不足/停氧时，能够及时使用备用氧，如氧气瓶等给传染病患者继续供氧** |  |  |  |  |  |  |
|  |  | **3.3.7不明原因晕倒应急处理** | **发现传染病患者、医护人员等晕倒时，能够及时采取安全有效的治疗和护理措施进行处理** |  |  |  |  |  |  |
|  |  | **3.3.8防护服破裂应急处理** | **防护服破裂时能够立即进行消毒处理，并采取预防性治疗** |  |  |  |  |  |  |
|  |  | **3.3.9物资短缺应急处理** | **防护物资短缺时，能够节约物资，寻找可以替代的物品** |  |  |  |  |  |  |
|  |  | **如有建议增加项目，请在下面空行填写（注：请判断其重要程度，并给出三级指标定义）** |  |  |  |  |  |  |  |
|  |  |  |  |  |  |  |  |  |  |
|  |  |  |  |  |  |  |  |  |  |
|  | **3.4综合救援能力** | **3.4.1评判性思维** | **在隔离病区工作时，能够敢于提出疑问，并进行分析、推理和判断** |  |  |  |  |  |  |
|  |  | **3.4.2科研能力** | **能够查阅文献，进行科研设计、撰写论文并分析数据** |  |  |  |  |  |  |
|  |  | **3.4.3病情观察及处置能力** | **能够密切观察传染病患者生命体征、意识等病情变化，及时采取措施** |  |  |  |  |  |  |
|  |  | **3.4.4自主学习能力** | **能够主动利用一切资源学习不同传染病的治疗、护理、预防等方面的知识** |  |  |  |  |  |  |
|  |  | **3.4.5检伤分诊能力** | **能够对传染病患者或疑似患者进行病情评估并分类处理** |  |  |  |  |  |  |
|  |  | **3.4.6文书书写能力** | **能够使用纸质记录或电子系统准确、无误的书写传染病患者护理记录** |  |  |  |  |  |  |
|  |  | **3.4.7教学能力** | **能够指导同事学习传染病相关的知识和技术；教会传染病患者做康复训练，对传染病患者进行健康宣教等** |  |  |  |  |  |  |
|  |  | **如有建议增加项目，请在下面空行填写（注：请判断其重要程度，并给出三级指标定义）** |  |  |  |  |  |  |  |
|  |  |  |  |  |  |  |  |  |  |
|  |  |  |  |  |  |  |  |  |  |
| **4.个人特质** | **4.1思想品质** | **4.1.1无私奉献精神** | **能够舍身忘己，默默为传染病患者奉献，不求回报** |  |  |  |  |  |  |
|  |  | **4.1.2慎独精神** | **在隔离病区无人监督时，能自觉按照道德规范做事** |  |  |  |  |  |  |
|  |  | **4.1.3吃苦耐劳精神** | **在隔离病区工作时，不怕条件艰苦，也不怕累** |  |  |  |  |  |  |
|  |  | **如有建议增加项目，请在下面空行填写（注：请判断其重要程度，并给出三级指标定义）** |  |  |  |  |  |  |  |
|  |  |  |  |  |  |  |  |  |  |
|  |  |  |  |  |  |  |  |  |  |
|  | **4.2综合素质** | **4.2.1身体素质** | **穿着厚重的防护装备时，能够坚持完成护理工作** |  |  |  |  |  |  |
|  |  | **4.2.2压力应对能力** | **面对隔离病区高强度、高挑战以及高传染性的工作，能够自动调节自身状态，从容应对** |  |  |  |  |  |  |
|  |  | **4.2.3责任心** | **能够自觉承担护理传染病患者或疑似患者的责任、履行护士的义务** |  |  |  |  |  |  |
|  |  | **4.2.4自信心** | **面对隔离病区中遇到的工作难题，相信自己能够解决** |  |  |  |  |  |  |
|  |  | **4.2.5乐观开朗** | **始终保持积极乐观的心态，调动传染病患者、同事的积极情绪** |  |  |  |  |  |  |
|  |  | **如有建议增加项目，请在下面空行填写（注：请判断其重要程度，并给出三级指标定义）** |  |  |  |  |  |  |  |
|  |  |  |  |  |  |  |  |  |  |
|  |  |  |  |  |  |  |  |  |  |

**表4 请选择您对以上条目的判断依据、影响程度及熟悉程度**

**在相应栏目内打钩“√”**

| **判断依据** | **影响程度** | | | | |
| --- | --- | --- | --- | --- | --- |
|  | **大** | **中** | | **小** | |
| **理论分析** |  |  | |  | |
| **实践经验** |  |  | |  | |
| **参考文献** |  |  | |  | |
| **直觉方面** |  |  | |  | |
| **您对本次调查内容的熟悉程度** | | | | | |
| **熟悉程度** | **很熟悉** | **比较熟悉** | **一般熟悉** | **不太熟悉** | **不熟悉** |
|  |  |  |  |  |  |

**填表结束，再次感谢您对本课题的支持与帮助！**

**祝您生活愉快！工作顺利！**
