# Supplementary material for: Construction of a competency evaluation index system for front-line nurses during the outbreak of major infectious diseases: A Delphi study
Source: PLoS One. 2022 Jul 1;17(7):e0270902. doi: 10.1371/journal.pone.0270902 (PMC9249240; doi:10.1371/journal.pone.0270902)
Supplement: S5 File — (DOCX) [file pone.0270902.s005.docx]

**突发重大传染病疫情下一线护理人员胜任力评价指标体系专家函询问卷（第二轮）**

尊敬的专家：

您好，首先衷心感谢您对本研究的支持和帮助以及您在第一轮函询中提出的宝贵意见！

经过第一轮对27名专家的函询，课题组成员在充分理解专家的意见和建议的基础上，结合文献查阅、承担过COVID-2019或其它传染病防疫工作医护人员的咨询结果，进行了积极认真地讨论，对各级指标进行了调整和修改，形成了第二轮问卷。为了让您更好地了解各位专家上一轮函询情况及指标修改、补充或增加的内容，我们在问卷函询表中增加了第一轮条目修改描述。

第二轮函询旨在对专家意见进行协调以及确定指标权重，请您对修改后的指标再次提出宝贵意见，这将对我们最终形成突发重大传染病疫情下一线护理人员胜任力评价指标体系具有重要意义。由于科研的时效性和研究进度的推进，恳请您于**一周之内**回复意见和建议。如果您对问卷有任何疑问，请随时联系我们。

衷心地感谢您的支持与指导！祝您工作顺利，身体健康！

重庆医科大学附属第二医院

导师：甘秀妮

研究生：白 雪

联系人：白雪 电话/微信：15761602836 邮箱：584454151@qq.com

**第一部分：突发重大传染病疫情下一线护理人员胜任力评价指标函询表**

**填表说明：**

**一、**此部分共包含3个函询表，其中：

表1：一级指标函询表；

表2：二级指标函询表；

表3：三级指标函询表；

二、请根据您自己的经验和知识，就每个指标的重要程度给予您的评分。

1.重要性评分：**非常重要=5分；比较重要=4分；一般=3分；不太重要=2分；不重要=1分**，请您在相应栏里打“√”或填写评分值。

2.如果您认为指标描述不准确或应删除，请在“修改或删减意见”栏内填写修改内容或注明“删除”。对于您认为还有我们未考虑到的需要增加的指标，请在“建议增加项目”空白栏内补充，并进行重要性评分。

**表1 一级指标函询表**

**注：重要性评分：非常重要=5分；比较重要=4分；一般=3分；不太重要=2分；不重要=1分**

| **一级指标** | **第二轮重要性评分** | | | | | **专家意见** | |
| --- | --- | --- | --- | --- | --- | --- | --- |
|  | **5** | **4** | **3** | **2** | **1** | **原第一轮条目** | **修改或删除意见** |
| **1.传染病知识体系** |  |  |  |  |  |  |  |
| **2.传染病相关护理技能** |  |  |  |  |  | **原为：传染病护理技术** |  |
| **3.传染病相关专业能力** |  |  |  |  |  | **原为：传染病应对能力** |  |
| **4.综合素质** |  |  |  |  |  | **原为：个人特质** |  |
| **如有建议增加项目，请在下面空行填写（注：请判断其重要程度）** |  |  |  |  |  |  |  |
|  |  |  |  |  |  |  |  |
|  |  |  |  |  |  |  |  |

**表2 二级指标函询表**

**注：重要性评分：非常重要=5分；比较重要=4分；一般=3分；不太重要=2分；不重要=1分**

| **一级指标** | **二级指标** | **第二轮重要性评分** | | | | | **专家意见** | |
| --- | --- | --- | --- | --- | --- | --- | --- | --- |
|  |  | **5** | **4** | **3** | **2** | **1** | **原第一轮条目** | **修改或删除意见** |
| **1.传染病知识体系** | **1.1传染病基础知识** |  |  |  |  |  |  |  |
|  | **1.2传染病相关知识** |  |  |  |  |  |  |  |
|  | **如有建议增加项目，请在下面空行填写（注：请判断其重要程度）** |  |  |  |  |  |  |  |
|  |  |  |  |  |  |  |  |  |
|  |  |  |  |  |  |  |  |  |
| **2.传染病相关护理技能** | **2.1传染病防护技术** |  |  |  |  |  |  |  |
|  | **2.2重症救护技术** |  |  |  |  |  |  |  |
|  | **2.3相关基础护理技术** |  |  |  |  |  | **原为：传染病防护下基础操作技术** |  |
|  | **如有建议增加项目，请在下面空行填写（注：请判断其重要程度）** |  |  |  |  |  |  |  |
|  |  |  |  |  |  |  |  |  |
|  |  |  |  |  |  |  |  |  |
| **3.传染病相关专业能力** | **3.1心理危机干预能力** |  |  |  |  |  |  |  |
|  | **3.2突发事件应急处置能力** |  |  |  |  |  | **原为：突发事件应对能力** |  |
|  | **3.3基础救援能力** |  |  |  |  |  | **原为：综合救援能力** |  |
|  | **如有建议增加项目，请在下面空行填写（注：请判断其重要程度）** |  |  |  |  |  |  |  |
|  |  |  |  |  |  |  |  |  |
|  |  |  |  |  |  |  |  |  |
| **4.综合素质** | **4.1思想品质** |  |  |  |  |  |  |  |
|  | **4.2身心素质** |  |  |  |  |  | **原为：综合素质** |  |
|  | **如有建议增加项目，请在下面空行填写（注：请判断其重要程度）** |  |  |  |  |  |  |  |
|  |  |  |  |  |  |  |  |  |
|  |  |  |  |  |  |  |  |  |

**表3 三级指标函询表**

**注：重要性评分：非常重要=5分；比较重要=4分；一般=3分；不太重要=2分；不重要=1分**

| **一级指标** | **二级指标** | **三级指标** | **三级指标定义** | **重要性评分** | | | | | **专家意见** | |
| --- | --- | --- | --- | --- | --- | --- | --- | --- | --- | --- |
|  |  |  |  | **5** | **4** | **3** | **2** | **1** | **原第一轮条目** | **修改或删除意见** |
| **1.传染病知识体系** | **1.1传染病基础知识** | **1.1.1传染病的概念及类型** | **掌握不同传染病的概念以及分类** |  |  |  |  |  |  |  |
|  |  | **1.1.2传染病的发病机制** | **掌握不同传染病的发生发展过程、组织损伤机制以及病理生理变化等** |  |  |  |  |  |  |  |
|  |  | **1.1.3传染病的流行病学特征** | **掌握不同传染病的流行性、季节性、地方性和在不同人群中的分布特点** |  |  |  |  |  |  |  |
|  |  | **1.1.4传染病的临床表现** | **掌握不同传染病的症状、体征等** |  |  |  |  |  |  |  |
|  |  | **1.1.5传染病的传播途径** | **掌握不同传染病的传播途径，如呼吸道传播、消化道传播、接触传播等** |  |  |  |  |  |  |  |
|  |  | **1.1.6传染病的预防措施** | **掌握不同传染病的预防方法及措施** |  |  |  |  |  |  |  |
|  |  | **1.1.7传染病的诊断标准** | **掌握不同传染病患者、疑似患者的诊断标准** |  |  |  |  |  |  |  |
|  |  | **1.1.8传染病的治疗及护理** | **掌握不同传染病的治疗原则和护理要点** |  |  |  |  |  |  |  |
|  |  | **1.1.9传染病的报告时间及报告流程** | **掌握不同传染病的上报时限和上报流程** |  |  |  |  |  | **原为：传染病的报告流程：掌握不同传染病的上报流程、传染病报告卡的填写** |  |
|  |  | **1.1.10传染病的相关检测** | **熟悉不同传染病的检测方法** |  |  |  |  |  | **原为：掌握不同传染病的检测方法** |  |
|  |  | **1.1.11传染病的应急处理流程** | **掌握不同传染病的应急处理流程。** |  |  |  |  |  | **新增** |  |
|  |  | **1.1.12传染病的病因** | **掌握不同传染病的发病原因** |  |  |  |  |  | **新增** |  |
|  |  | **如有建议增加项目，请在下面空行填写（注：请判断其重要程度，并给出三级指标定义）** |  |  |  |  |  |  |  |  |
|  |  |  |  |  |  |  |  |  |  |  |
|  |  |  |  |  |  |  |  |  |  |  |
|  | **1.2传染病相关知识** | **1.2.1法律、伦理知识** | **掌握传染病相关的法律法规，如《中华人民共和国传染病防治法》等；保护传染病患者的权利和隐私** |  |  |  |  |  |  |  |
|  |  | **1.2.2信息学知识** | **掌握隔离病区医疗系统、远程医学系统、传染病信息监测系统使用的相关知识，并能熟练应用** |  |  |  |  |  |  |  |
|  |  | **1.2.3合并慢性病的传染病复杂病例照护知识** | **具备对合并慢性病的传染病复杂病例的照护知识与技能，如高血压、冠心病、糖尿病等** |  |  |  |  |  | **新增** |  |
|  |  | **如有建议增加项目，请在下面空行填写（注：请判断其重要程度，并给出三级指标定义）** |  |  |  |  |  |  |  |  |
|  |  |  |  |  |  |  |  |  |  |  |
| **2.传染病相关护理技能** | **2.1传染病防护技术** | **2.1.1防护装备穿脱技术** | **能正确穿脱防护服、防护面罩、护目镜等防护装备** |  |  |  |  |  |  |  |
|  |  | **2.1.2手卫生** | **能按照规定正确执行手卫生** |  |  |  |  |  | **原为：能正确进行洗手、卫生手消毒、外科手消毒** |  |
|  |  | **2.1.3消毒灭菌技术** | **掌握各类常见传染病病原体的消毒灭菌方法** |  |  |  |  |  | **原为：掌握物理、化学消毒灭菌法，以及化学消毒剂的使用原则** |  |
|  |  | **2.1.4传染病医疗废物处理技术** | **掌握不同传染病医疗废物的处理方法与流程** |  |  |  |  |  | **新增** |  |
|  |  | **如有建议增加项目，请在下面空行填写（注：请判断其重要程度，并给出三级指标定义）** |  |  |  |  |  |  |  |  |
|  |  |  |  |  |  |  |  |  |  |  |
|  |  |  |  |  |  |  |  |  |  |  |
|  | **2.2重症救护技术** | **2.2.1心肺脑复苏技术** | **能正确为心脏骤停的传染病患者进行胸外心脏按压、人工辅助呼吸等** |  |  |  |  |  |  |  |
|  |  | **2.2.2心电监护仪使用及监测技术** | **掌握心电监护仪各指标的正常值及临床意义，识别常见心律失常** |  |  |  |  |  |  |  |
|  |  | **2.2.3营养支持技术** | **能在医生的指导下正确为传染病患者进行肠内外营养支持，掌握其适应症和禁忌症** |  |  |  |  |  | **原为：能正确为传染病患者进行肠内外营养支持，掌握其适应症和禁忌症** |  |
|  |  | **2.2.4血流动力学监测技术** | **能正确使用仪器对传染病患者血压，中心静脉压，肺动脉压，肺毛细血管楔压，心输出量等进行监测，掌握各指标的正常值及临床意义** |  |  |  |  |  |  |  |
|  |  | **2.2.5 CRRT技术** | **能正确为传染病患者实施CRRT，预防并发症发生，掌握其适应症和禁忌症，各指标的正常值及临床意义** |  |  |  |  |  |  |  |
|  |  | **2.2.6呼吸机使用及监测技术** | **能正确使用呼吸机，连接呼吸机管道，处理机器报警；掌握呼吸机不同模式的适应症和禁忌症，各指标的正常值及临床意义** |  |  |  |  |  |  |  |
|  |  | **2.2.7 ECMO使用及监测技术** | **能正确协助医生为传染病患者进行ECMO，处理机器报警；掌握其适应症和禁忌症，各指标的正常值及临床意义** |  |  |  |  |  |  |  |
|  |  | **2.2.8高流量吸氧装置使用及监测技术** | **能正确使用高流量吸氧装置，处理机器报警；掌握其适应症和禁忌症，各指标的正常值及临床意义** |  |  |  |  |  |  |  |
|  |  | **2.2.9除颤仪使用技术** | **能正确使用除颤仪，掌握其适应症和禁忌症，各指标的正常值及临床意义** |  |  |  |  |  |  |  |
|  |  | **2.2.10俯卧位通气技术** | **能正确帮助传染病患者进行俯卧位通气，掌握其适应症和禁忌症以及护理要点** |  |  |  |  |  |  |  |
|  |  | **2.2.11心电图机使用技术** | **能熟练使用心电图机，并对心电图结果进行分析** |  |  |  |  |  |  |  |
|  |  | **2.2.12微量泵/注射泵/输液泵使用技术** | **能正确使用微量泵/注射泵/输液泵，处理机器报警** |  |  |  |  |  |  |  |
|  |  | **2.2.13环甲膜穿刺术** | **能正确协助医生为传染病患者进行环甲膜穿刺，预防并发症发生** |  |  |  |  |  |  |  |
|  |  | **2.2.14气管插管/切开技术** | **能正确协助医生为传染病患者实施气管插管/切开，预防并发症发生** |  |  |  |  |  |  |  |
|  |  | **2.2.15简易呼吸器使用技术** | **能正确使用简易呼吸器辅助传染病患者呼吸** |  |  |  |  |  |  |  |
|  |  | **2.2.16气囊测压技术** | **能正确使用气囊测压仪进行气囊测压并适时调节** |  |  |  |  |  | **新增** |  |
|  |  | **2.2.17气道廓清技术** | **利用物理或机械方式作用于气流，帮助气管、支气管内的痰液排出或诱发咳嗽使痰液排出** |  |  |  |  |  | **新增** |  |
|  |  | **如有建议增加项目，请在下面空行填写（注：请判断其重要程度，并给出三级指标定义）** |  |  |  |  |  |  |  |  |
|  |  |  |  |  |  |  |  |  |  |  |
|  |  |  |  |  |  |  |  |  |  |  |
|  | **2.3相关基础护理技术** | **2.3.1标本采集、保存及运输技术** | **能正确采集传染病患者的痰、血液、咽拭子等标本，掌握各种标本的保存方法及运输方式** |  |  |  |  |  |  |  |
|  |  | **2.3.2血气分析技术** | **能正确使用血气分析机，掌握血气分析结果各指标的正常值及临床意义** |  |  |  |  |  |  |  |
|  |  | **2.3.3动静脉穿刺技术** | **能准确评估传染病患者的血管条件，进行外周和中心动静脉穿刺** |  |  |  |  |  |  |  |
|  |  | **如有建议增加项目，请在下面空行填写（注：请判断其重要程度，并给出三级指标定义）** |  |  |  |  |  |  |  |  |
|  |  |  |  |  |  |  |  |  |  |  |
|  |  |  |  |  |  |  |  |  |  |  |
| **3.传染病相关专业能力** | **3.1心理危机干预能力** | **3.1.1心理风险识别能力** | **掌握常见的心理评估量表，能够及时识别传染病患者的心理变化** |  |  |  |  |  |  |  |
|  |  | **3.1.2心理护理能力** | **能够运用心理学知识对传染病患者进行心理疏导，帮助患者恢复健康的心理状态** |  |  |  |  |  |  |  |
|  |  | **3.1.3人文关怀能力** | **能够尊重、关心传染病患者，以患者为中心** |  |  |  |  |  |  |  |
|  |  | **如有建议增加项目，请在下面空行填写（注：请判断其重要程度，并给出三级指标定义）** |  |  |  |  |  |  |  |  |
|  |  |  |  |  |  |  |  |  |  |  |
|  |  |  |  |  |  |  |  |  |  |  |
|  | **3.2突发事件应急处置能力** | **3.2.1针刺伤应急处理** | **能够正确处理伤口，上报相关部门，寻求医疗帮助** |  |  |  |  |  |  |  |
|  |  | **3.2.2血液/体液暴露应急处理** | **能够正确处理暴露在外的血液/体液，及时进行消毒** |  |  |  |  |  | **原为：能够正确处理暴露在外的血液/体液，及时进行处置** |  |
|  |  | **3.2.3停电应急处理** | **停电时能够冷静处理，及时启动备用电源** |  |  |  |  |  |  |  |
|  |  | **3.2.4火灾应急处理** | **发生火灾时能够及时、安全撤离传染病患者，把损害和影响降到最低** |  |  |  |  |  |  |  |
|  |  | **3.2.5自杀应急处理** | **能够及时劝解、抢救传染病患者，并向上级汇报** |  |  |  |  |  |  |  |
|  |  | **3.2.6中心供氧不足/停氧应急处理** | **中心供氧不足/停氧时，能够及时使用备用氧，如氧气瓶等给传染病患者继续供氧** |  |  |  |  |  |  |  |
|  |  | **3.2.7不明原因晕倒应急处理** | **发现传染病患者、医护人员等晕倒时，能够及时采取安全有效的治疗和护理措施进行处理** |  |  |  |  |  |  |  |
|  |  | **3.2.8防护装备破裂应急处理** | **防护装备破裂时能够立即进行消毒处理，并采取预防性治疗** |  |  |  |  |  | **原为：防护服破裂应急处理：防护服破裂时能够立即进行消毒处理，并采取预防性治疗** |  |
|  |  | **3.2.9物资短缺应急处理** | **防护物资短缺时，能够寻找达到防护级别的替代物品** |  |  |  |  |  | **原为：防护物资短缺时，能够节约物资，寻找可以替代的物品** |  |
|  |  | **如有建议增加项目，请在下面空行填写（注：请判断其重要程度，并给出三级指标定义）** |  |  |  |  |  |  |  |  |
|  |  |  |  |  |  |  |  |  |  |  |
|  |  |  |  |  |  |  |  |  |  |  |
|  | **3.3基础救援能力** | **3.3.1评判性思维** | **在隔离病区工作时，能够敢于提出疑问，并进行分析、推理和判断** |  |  |  |  |  |  |  |
|  |  | **3.3.2科研能力** | **能够查阅文献，进行科研设计、撰写论文并分析数据** |  |  |  |  |  |  |  |
|  |  | **3.3.3病情观察及处置能力** | **能够密切观察传染病患者生命体征、意识等病情变化，及时采取措施** |  |  |  |  |  |  |  |
|  |  | **3.3.4自主学习能力** | **能够主动利用一切资源学习不同传染病的治疗、护理、预防等方面的知识** |  |  |  |  |  |  |  |
|  |  | **3.3.5分诊转运能力** | **能够对传染病患者或疑似患者进行病情评估并分类处理** |  |  |  |  |  | **原为：检伤分诊能力** |  |
|  |  | **3.3.6文书书写能力** | **能够使用纸质记录或电子系统准确、无误的书写传染病患者护理记录** |  |  |  |  |  |  |  |
|  |  | **3.3.7教学能力** | **能够指导同事学习传染病相关的知识和技术；教会传染病患者做康复训练，对传染病患者进行健康宣教等** |  |  |  |  |  |  |  |
|  |  | **3.3.8沟通协调能力** | **能够与同事、传染病患者沟通顺畅，同时协调好医护、医患及护患关系** |  |  |  |  |  |  |  |
|  |  | **3.3.9团队协作能力** | **能够发挥团队精神，互帮互助，共同护理好传染病患者** |  |  |  |  |  |  |  |
|  |  | **3.3.10组织管理能力** | **能够管理好传染病患者、隔离病区，组织患者参加康复训练** |  |  |  |  |  | **原为：能够管理好传染病患者、隔离病区以及各种物资，组织患者参加康复训练** |  |
|  |  | **3.3.11外语能力** | **能阅读医疗仪器和防护物资上的外语信息，用外语与人交流** |  |  |  |  |  | **原为：外语知识** |  |
|  |  | **3.3.12工作经验** | **具备传染病科、ICU、呼吸科及参与重大传染病疫情救治等工作经验** |  |  |  |  |  | **原为：具备传染病、急危重症、呼吸科等工作经历** |  |
|  |  | **如有建议增加项目，请在下面空行填写（注：请判断其重要程度，并给出三级指标定义）** |  |  |  |  |  |  |  |  |
|  |  |  |  |  |  |  |  |  |  |  |
|  |  |  |  |  |  |  |  |  |  |  |
| **4.综合素质** | **4.1思想品质** | **4.1.1奉献精神** | **能够舍身忘己，默默为传染病患者奉献，不求回报** |  |  |  |  |  | **原为：无私奉献精神** |  |
|  |  | **4.1.2慎独精神** | **在隔离病区无人监督时，能自觉按照道德规范做事** |  |  |  |  |  |  |  |
|  |  | **4.1.3吃苦耐劳精神** | **在隔离病区工作时，不怕条件艰苦，也不怕累** |  |  |  |  |  |  |  |
|  |  | **如有建议增加项目，请在下面空行填写（注：请判断其重要程度，并给出三级指标定义）** |  |  |  |  |  |  |  |  |
|  |  |  |  |  |  |  |  |  |  |  |
|  |  |  |  |  |  |  |  |  |  |  |
|  | **4.2身心素质** | **4.2.1身体素质** | **穿着厚重的防护装备时，能够坚持完成护理工作** |  |  |  |  |  |  |  |
|  |  | **4.2.2压力应对能力** | **面对隔离病区高强度、高挑战以及高传染性的工作，能够自动调节自身状态，从容应对** |  |  |  |  |  |  |  |
|  |  | **4.2.3责任心** | **能够自觉承担护理传染病患者或疑似患者的责任、履行护士的义务** |  |  |  |  |  |  |  |
|  |  | **4.2.4自信心** | **面对隔离病区中遇到的工作难题，相信自己能够解决** |  |  |  |  |  |  |  |
|  |  | **4.2.5乐观开朗** | **始终保持积极乐观的心态，调动传染病患者、同事的积极情绪** |  |  |  |  |  |  |  |
|  |  | **如有建议增加项目，请在下面空行填写（注：请判断其重要程度，并给出三级指标定义）** |  |  |  |  |  |  |  |  |
|  |  |  |  |  |  |  |  |  |  |  |
|  |  |  |  |  |  |  |  |  |  |  |

**请选择您对以上条目的判断依据、影响程度及熟悉程度**

**在相应栏目内打钩“√”**

| **判断依据** | **影响程度** | | | | |
| --- | --- | --- | --- | --- | --- |
|  | **大** | **中** | | **小** | |
| **理论分析** |  |  | |  | |
| **实践经验** |  |  | |  | |
| **参考文献** |  |  | |  | |
| **直觉方面** |  |  | |  | |
| **您对本次调查内容的熟悉程度** | | | | | |
| **熟悉程度** | **很熟悉** | **比较熟悉** | **一般熟悉** | **不太熟悉** | **不熟悉** |
|  |  |  |  |  |  |

**填表结束，再次感谢您对本课题的支持与帮助！**

**祝您生活愉快！工作顺利！**

第二部分 层次分析法确定一、二、三级指标权重

**说明：**

本研究拟采用层次分析法（AHP）对各级评价指标进行权重的确定，即对各级指标项目进行两两重要性的比较，形成重要程度判断矩阵，利用 AHP 专用分析软件得到该评价体系各级指标的权重值。 填写说明：**重要性程度评分请按照表 1 中的计分方式进行取值**，并根据您自身的理解，**在空格处写上合适的比值，“－”处不需要填写。**

**表1 指标重要程度判断的取值方法**

| **相对重要程度（比较者:被比较者）** | **定义** | **说明** |
| --- | --- | --- |
| **1** | **同等重要** | **两个因素相比，“比较者”与“被比较者”同样重要** |
| **3** | **稍微重要** | **两个因素相比，“比较者”比“被比较者”稍微重要** |
| **5** | **明显重要** | **两个因素相比，“比较者”比“被比较者”明显重要** |
| **7** | **强烈重要** | **两个因素相比，“比较者”比“被比较者”强烈重要** |
| **9** | **绝对重要** | **两个因素相比，“比较者”比“被比较者”绝对重要** |
| **1/3** | **稍微不重要** | **两个因素相比，“比较者”比“被比较者”稍微不重要** |
| **1/5** | **明显不重要** | **两个因素相比，“比较者”比“被比较者”明显不重要** |
| **1/7** | **强烈不重要** | **两个因素相比，“比较者”比“被比较者”强烈不重要** |
| **1/9** | **绝对不重要** | **两个因素相比，“比较者”比“被比较者”绝对不重要** |
| **2、4、6、8或 1/2、1/4、1/6、1/8** |  | **表示相邻两标度之间的中值，如比较者:被比较者为4，指比较者:被比较者的重要程度介于稍微重要3和明显重要5的中间** |

**一、一级指标比较**

| **被比较者**  **比较者** | **1.传染病知识体系** | **2.传染病相关护理技能** | **3.传染病相关专业能力** | **4.综合素质** |
| --- | --- | --- | --- | --- |
| **1.传染病知识体系** | **－** |  |  |  |
| **2.传染病相关护理技能** | **－** | **－** |  |  |
| **3.传染病相关专业能力** | **－** | **－** | **－** |  |
| **4.综合素质** | **－** | **－** | **－** | **－** |

**二、二级指标比较**

**（1）传染病知识体系内部指标比较**

| **被比较者**  **比较者** | **1.1传染病基础知识** | **1.2传染病相关知识** |
| --- | --- | --- |
| **1.1传染病基础知识** | **－** |  |
| **1.2传染病相关知识** | **－** | **－** |

**（2）传染病相关护理技能内部指标比较**

| **被比较者**  **比较者** | **2.1传染病防护技术** | **2.2重症救护技术** | **2.3相关基础护理技术** |
| --- | --- | --- | --- |
| **2.1传染病防护技术** | **－** |  |  |
| **2.2重症救护技术** | **－** | **－** |  |
| **2.3相关基础护理技术** | **－** | **－** | **－** |

**（3）传染病相关专业能力内部指标比较**

| **被比较者**  **比较者** | **3.1心理危机干预能力** | **3.2突发事件应急处置能力** | **3.3基础救援能力** |
| --- | --- | --- | --- |
| **3.1心理危机干预能力** | **－** |  |  |
| **3.2突发事件应急处置能力** | **－** | **－** |  |
| **3.3基础救援能力** | **－** | **－** | **－** |

**（4）综合素质内部指标比较**

| **被比较者**  **比较者** | **4.1思想品质** | **4.2身心素质** |
| --- | --- | --- |
| **4.1思想品质** | **－** |  |
| **4.2身心素质** | **－** | **－** |

**三、三级指标比较**

**（1）传染病基础知识内部指标比较**

| **被比较者**  **比较者** | **1.1.1传染病的概念及类型** | **1.1.2传染病的发病机制** | **1.1.3传染病的流行病学特征** | **1.1.4传染病的临床表现** | **1.1.5传染病的传播途径** | **1.1.6传染病的预防措施** | **1.1.7传染病的诊断标准** | **1.1.8传染病的治疗及护理** | **1.1.9传染病的报告时间及报告流程** | **1.1.10传染病的相关检测** | **1.1.11传染病的应急处理流程** | **1.1.12传染病的病因** |
| --- | --- | --- | --- | --- | --- | --- | --- | --- | --- | --- | --- | --- |
| **1.1.1传染病的概念及类型** | **－** |  |  |  |  |  |  |  |  |  |  |  |
| **1.1.2传染病的发病机制** | **－** | **－** |  |  |  |  |  |  |  |  |  |  |
| **1.1.3传染病的流行病学特征** | **－** | **－** | **－** |  |  |  |  |  |  |  |  |  |
| **1.1.4传染病的临床表现** | **－** | **－** | **－** | **－** |  |  |  |  |  |  |  |  |
| **1.1.5传染病的传播途径** | **－** | **－** | **－** | **－** | **－** |  |  |  |  |  |  |  |
| **1.1.6传染病的预防措施** | **－** | **－** | **－** | **－** | **－** | **－** |  |  |  |  |  |  |
| **1.1.7传染病的诊断标准** | **－** | **－** | **－** | **－** | **－** | **－** | **－** |  |  |  |  |  |
| **1.1.8传染病的治疗及护理** | **－** | **－** | **－** | **－** | **－** | **－** | **－** | **－** |  |  |  |  |
| **1.1.9传染病的报告时间及报告流程** | **－** | **－** | **－** | **－** | **－** | **－** | **－** | **－** | **－** |  |  |  |
| **1.1.10传染病的相关检测** | **－** | **－** | **－** | **－** | **－** | **－** | **－** | **－** | **－** | **－** |  |  |
| **1.1.11传染病的应急处理流程** | **－** | **－** | **－** | **－** | **－** | **－** | **－** | **－** | **－** | **－** | **－** |  |
| **1.1.12传染病的病因** | **－** | **－** | **－** | **－** | **－** | **－** | **－** | **－** | **－** | **－** | **－** | **－** |

1. **传染病相关知识内部指标比较**

| **被比较者**  **比较者** | **1.2.1法律、伦理知识** | **1.2.2信息学知识** | **1.2.3合并慢性病的传染病复杂病例照护知识** |
| --- | --- | --- | --- |
| **1.2.1法律、伦理知识** | **－** |  |  |
| **1.2.2信息学知识** | **－** | **－** |  |
| **1.2.3合并慢性病的传染病复杂病例照护知识** | **－** | **－** | **－** |

**（3）传染病防护技术内部指标比较**

| **被比较者**  **比较者** | **2.1.1防护装备穿脱技术** | **2.1.2手卫生** | **2.1.3消毒灭菌技术** | **2.1.4传染病医疗废物处理技术** |
| --- | --- | --- | --- | --- |
| **2.1.1防护装备穿脱技术** | **－** |  |  |  |
| **2.1.2手卫生** | **－** | **－** |  |  |
| **2.1.3消毒灭菌技术** | **－** | **－** | **－** |  |
| **2.1.4传染病医疗废物处理技术** | **－** | **－** | **－** | **－** |

**（4）重症救护技术内部指标比较**

| **被比较者**  **比较者** | **2.2.1心肺脑复苏技术** | **2.2.2心电监护仪使用及监测技术** | **2.2.3营养支持技术** | **2.2.4血流动力学监测技术** | **2.2.5 CRRT技术** | **2.2.6呼吸机使用及监测技术** | **2.2.7 ECMO使用及监测技术** | **2.2.8高流量吸氧装置使用及监测技术** | **2.2.9除颤仪使用技术** | **2.2.10俯卧位通气技术** | **2.2.11心电图机使用技术** | **2.2.12微量泵/注射泵/输液泵使用技术** | **2.2.13环甲膜穿刺术** | **2.2.14气管插管/切开技术** | **2.2.15简易呼吸器使用技术** | **2.2.16气囊测压技术** | **2.2.17气道廓清技术** |
| --- | --- | --- | --- | --- | --- | --- | --- | --- | --- | --- | --- | --- | --- | --- | --- | --- | --- |
| **2.2.1心肺脑复苏技术** | **－** |  |  |  |  |  |  |  |  |  |  |  |  |  |  |  |  |
| **2.2.2心电监护仪使用及监测技术** | **－** | **－** |  |  |  |  |  |  |  |  |  |  |  |  |  |  |  |
| **2.2.3营养支持技术** | **－** | **－** | **－** |  |  |  |  |  |  |  |  |  |  |  |  |  |  |
| **2.2.4血流动力学监测技术** | **－** | **－** | **－** | **－** |  |  |  |  |  |  |  |  |  |  |  |  |  |
| **2.2.5 CRRT技术** | **－** | **－** | **－** | **－** | **－** |  |  |  |  |  |  |  |  |  |  |  |  |
| **2.2.6呼吸机使用及监测技术** | **－** | **－** | **－** | **－** | **－** | **－** |  |  |  |  |  |  |  |  |  |  |  |
| **2.2.7 ECMO使用及监测技术** | **－** | **－** | **－** | **－** | **－** | **－** | **－** |  |  |  |  |  |  |  |  |  |  |
| **2.2.8高流量吸氧装置使用及监测技术** | **－** | **－** | **－** | **－** | **－** | **－** | **－** | **－** |  |  |  |  |  |  |  |  |  |
| **2.2.9除颤仪使用技术** | **－** | **－** | **－** | **－** | **－** | **－** | **－** | **－** | **－** |  |  |  |  |  |  |  |  |
| **2.2.10俯卧位通气技术** | **－** | **－** | **－** | **－** | **－** | **－** | **－** | **－** | **－** | **－** |  |  |  |  |  |  |  |
| **2.2.11心电图机使用技术** | **－** | **－** | **－** | **－** | **－** | **－** | **－** | **－** | **－** | **－** | **－** |  |  |  |  |  |  |
| **2.2.12微量泵/注射泵/输液泵使用技术** | **－** | **－** | **－** | **－** | **－** | **－** | **－** | **－** | **－** | **－** | **－** | **－** |  |  |  |  |  |
| **2.2.13环甲膜穿刺术** | **－** | **－** | **－** | **－** | **－** | **－** | **－** | **－** | **－** | **－** | **－** | **－** | **－** |  |  |  |  |
| **2.2.14气管插管/切开技术** | **－** | **－** | **－** | **－** | **－** | **－** | **－** | **－** | **－** | **－** | **－** | **－** | **－** | **－** |  |  |  |
| **2.2.15简易呼吸器使用技术** | **－** | **－** | **－** | **－** | **－** | **－** | **－** | **－** | **－** | **－** | **－** | **－** | **－** | **－** | **－** |  |  |
| **2.2.16气囊测压技术** | **－** | **－** | **－** | **－** | **－** | **－** | **－** | **－** | **－** | **－** | **－** | **－** | **－** | **－** | **－** | **－** |  |
| **2.2.17气道廓清技术** | **－** | **－** | **－** | **－** | **－** | **－** | **－** | **－** | **－** | **－** | **－** | **－** | **－** | **－** | **－** | **－** | **－** |

**（5）相关基础护理技术内部指标比较**

| **被比较者**  **比较者** | **2.3.1标本采集、保存及运输技术** | **2.3.2血气分析技术** | **2.3.3动静脉穿刺技术** |
| --- | --- | --- | --- |
| **2.3.1标本采集、保存及运输技术** | **－** |  |  |
| **2.3.2血气分析技术** | **－** | **－** |  |
| **2.3.3动静脉穿刺技术** | **－** | **－** | **－** |

**（6）心理危机干预能力内部指标比较**

| **被比较者**  **比较者** | **3.1.1心理风险识别能力** | **3.1.2心理护理能力** | **3.1.3人文关怀能力** |
| --- | --- | --- | --- |
| **3.1.1心理风险识别能力** | **－** |  |  |
| **3.1.2心理护理能力** | **－** | **－** |  |
| **3.1.3人文关怀能力** | **－** | **－** | **－** |

**（7）突发事件应急处置能力内部指标比较**

| **被比较者**  **比较者** | **3.2.1针刺伤应急处理** | **3.2.2血液/体液暴露应急处理** | **3.2.3停电应急处理** | **3.2.4火灾应急处理** | **3.2.5自杀应急处理** | **3.2.6中心供氧不足/停氧应急处理** | **3.2.7不明原因晕倒应急处理** | **3.2.8防护装备破裂应急处理** | **3.2.9物资短缺应急处理** |
| --- | --- | --- | --- | --- | --- | --- | --- | --- | --- |
| **3.2.1针刺伤应急处理** | **－** |  |  |  |  |  |  |  |  |
| **3.2.2血液/体液暴露应急处理** | **－** | **－** |  |  |  |  |  |  |  |
| **3.2.3停电应急处理** | **－** | **－** | **－** |  |  |  |  |  |  |
| **3.2.4火灾应急处理** | **－** | **－** | **－** | **－** |  |  |  |  |  |
| **3.2.5自杀应急处理** | **－** | **－** | **－** | **－** | **－** |  |  |  |  |
| **3.2.6中心供氧不足/停氧应急处理** | **－** | **－** | **－** | **－** | **－** | **－** |  |  |  |
| **3.2.7不明原因晕倒应急处理** | **－** | **－** | **－** | **－** | **－** | **－** | **－** |  |  |
| **3.2.8防护装备破裂应急处理** | **－** | **－** | **－** | **－** | **－** | **－** | **－** | **－** |  |
| **3.2.9物资短缺应急处理** | **－** | **－** | **－** | **－** | **－** | **－** | **－** | **－** | **－** |

1. **基础救援能力内部指标比较**

| **被比较者**  **比较者** | **3.3.1评判性思维** | **3.3.2科研能力** | **3.3.3病情观察及处置能力** | **3.3.4自主学习能力** | **3.3.5分诊转运能力** | **3.3.6文书书写能力** | **3.3.7教学能力** | **3.3.8沟通协调能力** | **3.3.9团队协作能力** | **3.3.10组织管理能力** | **3.3.11外语能力** | **3.3.12工作经验** |
| --- | --- | --- | --- | --- | --- | --- | --- | --- | --- | --- | --- | --- |
| **3.3.1评判性思维** | **－** |  |  |  |  |  |  |  |  |  |  |  |
| **3.3.2科研能力** | **－** | **－** |  |  |  |  |  |  |  |  |  |  |
| **3.3.3病情观察及处置能力** | **－** | **－** | **－** |  |  |  |  |  |  |  |  |  |
| **3.3.4自主学习能力** | **－** | **－** | **－** | **－** |  |  |  |  |  |  |  |  |
| **3.3.5分诊转运能力** | **－** | **－** | **－** | **－** | **－** |  |  |  |  |  |  |  |
| **3.3.6文书书写能力** | **－** | **－** | **－** | **－** | **－** | **－** |  |  |  |  |  |  |
| **3.3.7教学能力** | **－** | **－** | **－** | **－** | **－** | **－** | **－** |  |  |  |  |  |
| **3.3.8沟通协调能力** | **－** | **－** | **－** | **－** | **－** | **－** | **－** | **－** |  |  |  |  |
| **3.3.9团队协作能力** | **－** | **－** | **－** | **－** | **－** | **－** | **－** | **－** | **－** |  |  |  |
| **3.3.10组织管理能力** | **－** | **－** | **－** | **－** | **－** | **－** | **－** | **－** | **－** | **－** |  |  |
| **3.3.11外语能力** | **－** | **－** | **－** | **－** | **－** | **－** | **－** | **－** | **－** | **－** | **－** |  |
| **3.3.12工作经验** | **－** | **－** | **－** | **－** | **－** | **－** | **－** | **－** | **－** | **－** | **－** | **－** |

**（9）思想品质内部指标比较**

| **被比较者**  **比较者** | **4.1.1奉献精神** | **4.1.2慎独精神** | **4.1.3吃苦耐劳精神** |
| --- | --- | --- | --- |
| **4.1.1奉献精神** | **－** |  |  |
| **4.1.2慎独精神** | **－** | **－** |  |
| **4.1.3吃苦耐劳精神** | **－** | **－** | **－** |

**（10）身心素质内部指标比较**

| **被比较者**  **比较者** | **4.2.1身体素质** | **4.2.2压力应对能力** | **4.2.3责任心** | **4.2.4自信心** | **4.2.5乐观开朗** |
| --- | --- | --- | --- | --- | --- |
| **4.2.1身体素质** | **－** |  |  |  |  |
| **4.2.2压力应对能力** | **－** | **－** |  |  |  |
| **4.2.3责任心** | **－** | **－** | **－** |  |  |
| **4.2.4自信心** | **－** | **－** | **－** | **－** |  |
| **4.2.5乐观开朗** | **－** | **－** | **－** | **－** | **－** |
